# Recent Historical Migrations Have Shaped the Gene Pool of Arabs and Berbers in North Africa

**DOI:** 10.1093/molbev/msw218

**Published:** 2016-10-15

**Authors:** Lara R. Arauna, Javier Mendoza-Revilla, Alex Mas-Sandoval, Hassan Izaabel, Asmahan Bekada, Soraya Benhamamouch, Karima Fadhlaoui-Zid, Pierre Zalloua, Garrett Hellenthal, David Comas

**Affiliations:** 1 Departament de Ciències Experimentals i de la Salut, Institute of Evolutionary Biology (CSIC-UPF), Universitat Pompeu Fabra, Barcelona, Spain; 2 Genetics Institute, University College London, London, United Kingdom; 3 Departamento de Genética, Instituto de Biociências, Universidade Federal do Rio Grande do Sul, Porto Alegre, Brazil; 4 Laboratoire de Biologie Cellulaire et Génétique Moléculaire (LBCGM), Université IBNZOHR, Agadir, Morocco; 5 Département de Biotechnologie, Faculté des Sciences de la Nature et de la Vie, Université Oran 1 (Ahmad Ben Bella), Oran, Algeria; 6 Laboratoire de Génetique, Immunologie et Pathologies Humaines, Faculté des Sciences de Tunis, Campus Universitaire El Manar II, Université El Manar, Tunis, Tunisia; 7 The Lebanese American University, Chouran, Beirut, Lebanon

**Keywords:** population genetics, North Africa, genome wide SNPs, Berbers, haplotype, admixture

## Abstract

North Africa is characterized by its diverse cultural and linguistic groups and its genetic heterogeneity. Genomic data has shown an amalgam of components mixed since pre-Holocean times. Though no differences have been found in uniparental and classical markers between Berbers and Arabs, the two main ethnic groups in the region, the scanty genomic data available have highlighted the singularity of Berbers. We characterize the genetic heterogeneity of North African groups, focusing on the putative differences of Berbers and Arabs, and estimate migration dates. We analyze genome-wide autosomal data in five Berber and six Arab groups, and compare them to Middle Easterns, sub-Saharans, and Europeans. Haplotype-based methods show a lack of correlation between geographical and genetic populations, and a high degree of genetic heterogeneity, without strong differences between Berbers and Arabs. Berbers enclose genetically diverse groups, from isolated endogamous groups with high autochthonous component frequencies, large homozygosity runs and low effective population sizes, to admixed groups with high frequencies of sub-Saharan and Middle Eastern components. Admixture time estimates show a complex pattern of recent historical migrations, with a peak around the 7th century C.E. coincident with the Arabization of the region; sub-Saharan migrations since the 1st century B.C. in agreement with Roman slave trade; and a strong migration in the 17th century C.E., coincident with a huge impact of the trans-Atlantic and trans-Saharan trade of sub-Saharan slaves in the Modern Era. The genetic complexity found should be taken into account when selecting reference groups in population genetics and biomedical studies.

## Introduction

North African human populations are the result of an amalgam of migrations due to their strategic location at a crossroads of three continents: limited to the south by the Sahara desert, which has acted as a permeable barrier with the rest of the African continent; the Mediterranean basin in the coast, which has allowed the transit of maritime civilizations from Europe; and the connection to the Middle East by the Arabian Peninsula and the Sinai, which has permitted constant migrations by ground. The human presence in North Africa dates back 130–190 Kya (Smith et al. 2007) and different cultures are identified in archaeological records, since the local Aterian, followed by the Iberomaurusian during the Holocene, and the Capsian culture that arose before the Neolithic (Hunt et al. 2010; Barton et al. 2013; Scerri 2013). The population continuity or replacement of these ancient cultures is under debate, although events of replacement have been supported by genetic and archaeological studies (Irish 2000; Henn et al. 2012), suggesting that the first Paleolithic settlers might not be the direct ancestors of extant North African populations.

Historical records affirm that North Africa was populated by different groups supposed to be the ancestors of the current Berber peoples (Amazigh), by the arrival of Phoenicians in the second millennium B.C., and the posterior conquest of the area by the Romans. The Roman control persisted until the 5th century C.E., although non-Romanized Berber tribes persisted all over the region. The Arab expansion started in the Arabian Peninsula in the 7th century C.E. through Egypt and expanded until reaching the westernmost part of North Africa (i.e., the Maghreb). The rule of the Arab dynasties ended with a decline in the 16th century, when the Ottoman Empire took control of the region until the colonization during the 18th and 19th centuries by European countries (Newman 1995). The complexity of these known (and unknown) historical migrations might have left genetic traces in North African populations that could be reconstructed by, e.g., recent haplotype-based approaches (Lawson et al. 2012; Hellenthal et al. 2014).

In addition to the migration complexity found in North Africa, a cultural diversity is characterized by the presence of two main branches of languages, both included in the Afro-Asiatic family (Ruhlen 1991): the Arab, introduced in the region from the Middle East during the Arab expansion together with Islam; and the Berber languages, which are composed by many different languages and dialects. Nowadays, Berbers are identified by the use of a Berber language, and are considered the ancestral peoples of North Africa. Despite the pivotal importance of Berbers for the knowledge of North African history, limited genetic analyses have been performed beyond the study of uniparental markers, which showed high heterogeneity in Berber samples, presence of autochthonous lineages (i.e., mitochondrial U6 and M1; and Y-chromosome E-M78 and E-M81 haplogroups), and lack of differentiation between Berber and Arab groups (Bosch et al. 2001; Plaza et al. 2003; Arredi et al. 2004; Coudray et al. 2009; Fadhlaoui-Zid et al. 2011b; Fadhlaoui-Zid et al. 2013; Bekada et al. 2015).

There is scanty genome-wide data in North African groups that can help to unravel the complexity of human migrations in the area. The pioneer study of ∼900 K SNPs by Henn et al. (2012) showed an amalgam of ancestral components (i.e., sub-Saharan, Maghrebi, European, and Middle Eastern) in North African groups. The presence of these components in the region, estimated by Fst methods and maximum likelihood approaches based on the analysis of tract lengths, suggested a back-to-Africa in pre-Holocene times (>12,000 ya) and recent sub-Saharan historical migrations, although the time estimates of the relevant Middle Eastern component in North Africa was not addressed. In addition, the analysis of North African samples showed a differentiation of the single Berber sample analyzed (characterized by high frequency of the autochthonous Maghrebi component) compared with the rest of Arab samples in the study, which challenged the hypothesis of lack of genetic differentiation shown by uniparental markers. However, the inclusion of a single Berber sample in the study limits the conclusion regarding the differentiation between Arab and Berber groups, since the differentiation of the Berber sample analyzed might be due to a genetic singularity of Berbers or to isolation and drift of this specific sample. This lack of knowledge prevents from dissecting the autochthonous genetic North African component. In addition to this study, the inclusion in the Human Genome Diversity Panel (HGDP-CEPH) (Cann et al. 2002) of a Berber isolate from Algeria, the Mozabite, has limited the complexity of North Africa (and even sometimes the Middle East) to a single proxy, which ignores the genetic heterogeneity of the region.

To explore the genetic complexity of North African groups and correlate it with the cultural diversity present in the area, we have genotyped ∼900 K SNPs in four additional Berber groups from Morocco, Algeria, and Tunisia. We combine this data with some published and some new reference panels, and apply haplotype-based approaches to characterize the ancestry components of North African groups and estimate admixture dates of the migrations that have shaped the genetic landscape of the region. Our results highlight the idea of a heterogeneous genetic landscape in North Africa due to recent demographic events, which should be taken into account when performing biomedical studies.

## Results

**Fig. 1.**
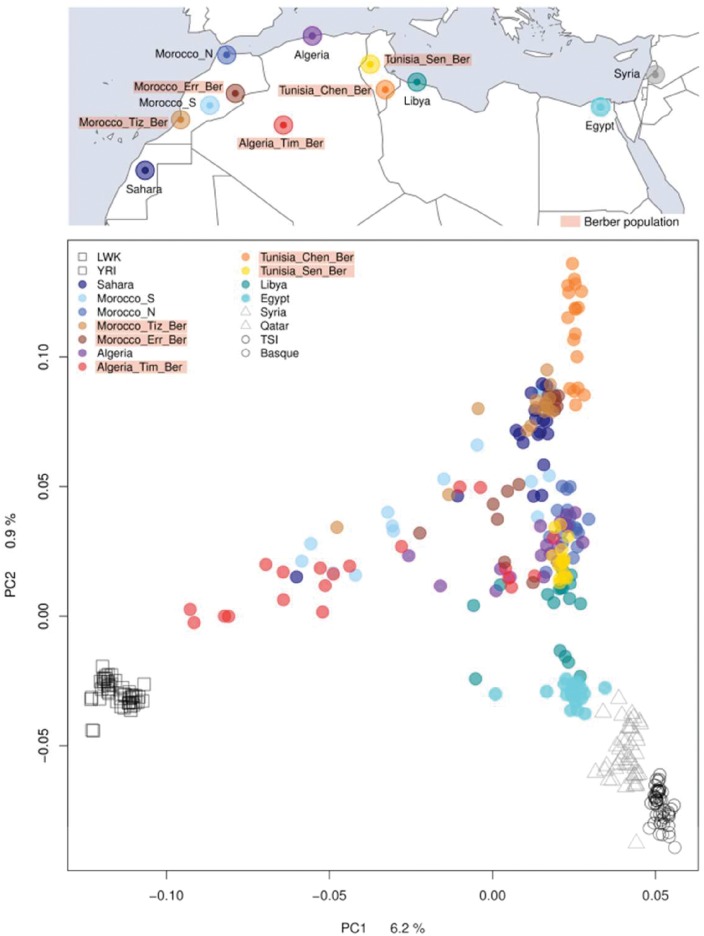
Population Structure in North Africa. Principal component analysis (PCA) and sample location. Berber-speaking samples are highlighted in the map and PCA legend.

**Fig. 2.**
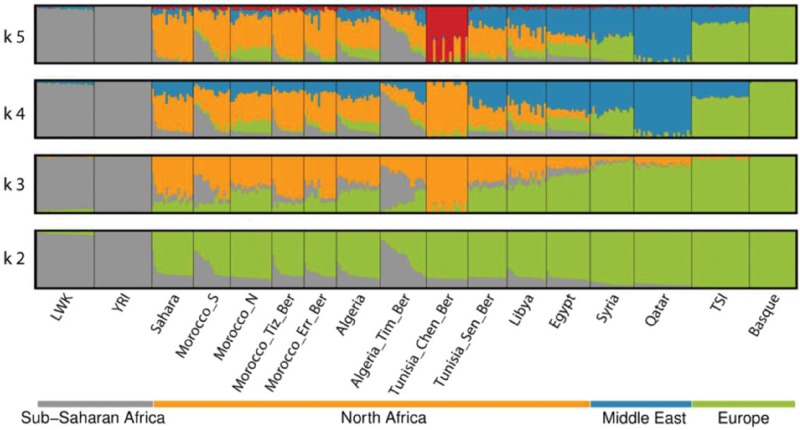
ADMIXTURE plots with North African and surrounding populations. ADMIXTURE plots from *k* = 2 to *k* = 5 of the high density dataset are shown.

The previously described complex and heterogeneous genetic structure of North African populations is shown in our Principal Component (PC) (fig. 1 and supplementary fig. S1, Supplementary Material online) and ADMIXTURE analyses (fig. 2). North African samples show a mixed pattern of components in the unsupervised ADMIXTURE analysis. The lowest-cross validation error considering the high SNP density dataset is found when three ancestral components are considered (*k* = 3) (supplementary fig. S2, Supplementary Material online). The three clusters found correspond to a sub-Saharan ancestral component (gray); a component found in North African populations (yellow), pointing to a North African ancestral component; and a third component, predominantly European (green). When an additional component is added (*k* = 4), this “European component” is divided into one more linked to European populations, and another predominant in Middle East (blue). North African individuals, regardless of their Berber or Arab language affiliation, show a mixture of these components. These four components are correlated with geography (i.e., longitude), with the North African (*r* = −0.868, *P* = 3.036 × 10^−^^6^) and sub-Saharan (*r* = −0.567, *P* = 0.014) components significantly increased in the West, whereas the European (*r* = 0.6, *P* = 0.008) and Middle Eastern (*r* = 0.925, *P* = 3.951 × 10^−^^8^) components increased in the East (supplementary fig. S3, Supplementary Material online). Additional components show substructure in North African as well as sub-Saharan African and Middle Eastern samples (supplementary fig. S4, Supplementary Material online). These ADMIXTURE results agree with what is shown in the PC plot (fig. 1), which separates sub-Saharan individuals and European and Middle Eastern in opposite edges of the first PC, whereas all North African samples remain in between. This first PC is highly correlated with the frequency of the sub-Saharan component in the ADMIXTURE analysis (at *k* = 4; *r* = 0.928, *P* = <2.2 × 10^−^^16^) (supplementary fig. S5, Supplementary Material online). The second PC separates a Tunisian Berber (Chenini) sample in one edge, whereas the North African individuals are dispersed in the PC plot, suggesting a high heterogeneity among groups and in some cases even within groups, which contrast with the rest of populations (i.e., Yoruba, Basque, Tuscan, and Syrians) that show high genetic homogeneity. This second PC is highly correlated with the North African component found on the ADMIXTURE analysis (at *k* = 4; *r* = 0.981, *P* = <2.2 × 10^−^^16^) (supplementary fig. S5, Supplementary Material online).

In the PCA, the Berber-speaking individuals cluster with the rest of North African non-Berber samples without making differentiable clusters. The two groups of Berbers from Morocco (Tiznit and Errachidia) lie intermingled with non-Berber Moroccan samples; and Tunisian Berbers from Sened lie within their geographically non-Berber closer relatives, the Libyans, and the Algerians. The Algerian Berbers from Timimoun show a higher diversity, making a gradient towards sub-Saharan African samples and exhibit a higher frequency of the sub-Saharan ancestral component in the ADMIXTURE analysis. Finally, Tunisian Berbers from Chenini, which were the only Berber-speaking group included in a previous North African genome-wide analysis (Henn et al. 2012), form a distinct cluster in both PCA and ADMIXTURE that separates them from other Berber samples, perhaps indicative of relatively high levels of recent interbreeding in this group as we explore below.

**Fig. 3.**
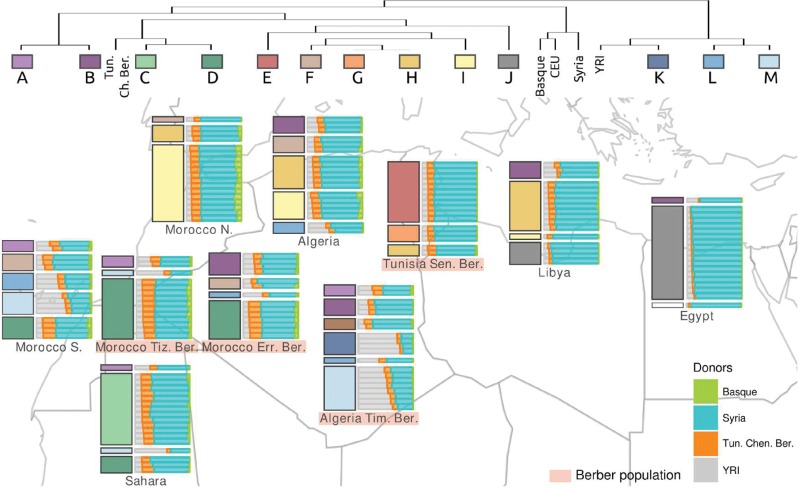
Haplotype sharing and FineStructure cluster distribution in North Africa. The right horizontal barplots in each geographic location show individual proportions of inferred haplotype sharing with Yoruba, Tunisian Berbers from Chenini, Syrians, and Basques, estimated using a mixture model that incorporates ChromoPainter results (colors according to the bottom right legend in the figure). Within each geographic location, left vertical barplots show the proportion of individuals from each population assigned to each of 13 North African fineSTRUCTURE genetic clusters (colors match the fineSTRUCTURE simplified dendrogram represented at the top of the figure).

In order to analyze in depth the complex structure and heterogeneous genetic patterns of North African populations, we explored patterns of haplotype sharing using ChromoPainter and fineSTRUCTURE (Lawson et al. 2012). Both the Chromo Painter coancestry matrix (supplementary figs. S6 and S7, Supplementary Material online), which measures the amount of haplotype sharing among groups, and the fineSTRUCTURE-inferred tree that clusters genetically similar individuals (supplementary fig. S7, Supplementary Material online), reinforce the complex structure of North African populations. Based on our fineSTRUCTURE results, we classified our 190 North African individuals into 14 clusters (fig. 3). Current North African geographical samples do not form homogeneous genetic populations, with the single exception of Tunisian Berbers from Chenini (cluster Tun.Chen.Ber. in fig. 3), contrasting with the genetic homogeneity shown by surrounding populations (clusters Basque, CEU, Syria, and YRI in fig. 3). Despite the genetic heterogeneity in North Africa, some geographical or population structure can be detected among the fineSTRUCTURE-inferred clusters. Clusters L and M that branch close to Yoruba are found in the Southwest; clusters B, H, and I are mainly restricted to the northern coast; whereas cluster J is restricted to the East and present in Libya and Egypt. Finally, some clusters are specific to some geographical populations such as cluster C in Western Sahara; clusters E and G in Tunisian Berbers from Sened; and cluster K in Algerian Berbers. It is worth noting that the distribution of clusters does not correlate with the ethno-linguistic affiliation of the samples, i.e. no general or common “Berber” or “Arab” cluster is found.

As shown in the description of the genetic clusters above, the geographical North African populations contain a mixture of individuals with different genetic histories. Representing each North African individual’s ChromoPainter inferred haplotype sharing patterns as a mixture of that from four genetically different groups (Yoruba, Tunisian Berbers from Chenini, Syrians, and Basques) helps to illustrate this substructure (fig. 3). Under this haplotype sharing distribution, the fineSTRUCTURE clusters described above vary mainly in their proportions of Middle Eastern and sub-Saharan ancestry; in some cases (such as Tunisian Berbers from Sened) there are clusters with similar ancestry proportions that might differ by genetic drift as a result of isolation and interbreeding (supplementary fig. S8, Supplementary Material online). The comparison of the ancestry proportions of the X chromosome and autosomes showed a significant gene-flow bias in three North African clusters (I, M, and D) containing more than 10 individuals (supplementary fig. S9, Supplementary Material online). Sub-Saharan African ancestry was higher in the autosomes relative to the X chromosome on clusters I and D, though the absolute difference was small (<3.5%). Significantly higher Tunisian Chenini-like ancestry was also found in the autosomes in cluster D, as well as significantly higher Middle Eastern ancestry in the autosomes in cluster M with differences of 3.9% and 12.2%, respectively. North African clusters K, A, and G also showed significant gene-flow bias, although these clusters had the lowest sample sizes (<7 individuals), which could have contributed to greater variation in the ancestry estimations, making these comparisons less reliable. It is also interesting to note that, although not always significant, there is consistently higher Tunisian Chenini-like ancestry on the autosomes in all North African clusters. The exception to this is on cluster J, which consisted uniquely of individuals from Egypt and Libya (populations with the highest Middle Eastern ancestry, fig. 1). Another notable finding was the higher Basque-like ancestry on the X chromosome on all North African clusters except on clusters E, I, and J. To assess the reliability of the difference between the X chromosome and the autosomes, we compared the ancestry estimations for chromosomes 1–6 in turn to all remaining autosomes (supplementary table S4, Supplementary Material online). We found that the comparison of chromosome 1 and 2 did not show any significant differences, except in the clusters with a sample size lower than 10 individuals, but that the comparisons of chromosomes 3–6 did show significant differences even in clusters with higher sample sizes. Since chromosome X had the highest number of SNPs in our data, we think our X chromosome comparisons are not biased due to the difference in the number of SNPs between the X chromosome and the autosomes. However, results can still be affected by lower sample size, and we caution against making comparisons when using small sample sizes.

**Fig. 4.**
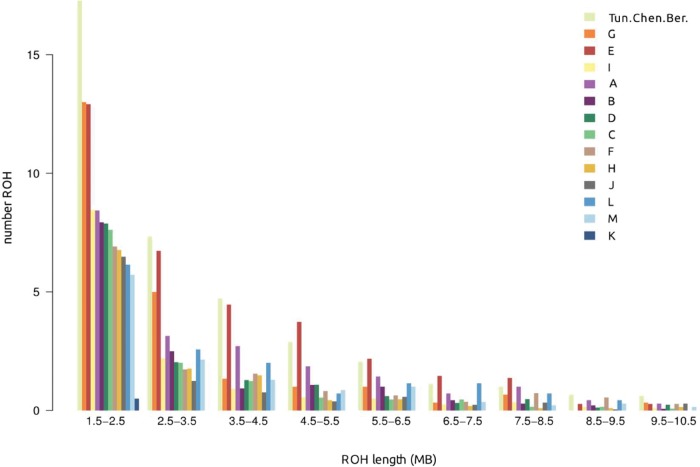
Runs of homozygosity (ROH) in North Africa. Barplot showing the ROH estimated for each genetic cluster starting at 1.5 MB.

Since Berber-speaking groups have been suggested to be descendant of isolated, fragmented and endogamous populations (Fadhlaoui-Zid et al. 2011a; Bekada et al. 2015), an analysis of runs of homozygosity (ROH) that are indicative of recent intermixing was performed within each population (Kirin et al. 2010; Stevens et al. 2011). The cluster Tun.Chen.Ber (which represent uniquely and entirely the Berber population of Tunisia Chenini), and the clusters E and G (which are only present in the Berber population of Tunisia Sened) present higher number of ROH in all length categories (fig. 4 and supplementary fig. S10, Supplementary Material online), indicative of recent inbreeding. However, other Berber-speaking groups exhibit similar ROH patterns as non-Berber groups, disclaiming the general view of Berber-speaking populations as being more interbred and isolated compared with other North African groups. In addition to the ROH analysis, effective population sizes (Ne) were estimated for each geographical population (supplementary fig. S11, Supplementary Material online). In agreement with our previous results, Ne is very low in Tunisian Berbers from Chenini (3366.50, standard error = 74.42), and in Tunisian Berbers from Sened (6340.9, standard error = 117.9) relative to the rest of North African samples. However, some of the Ne estimates might be influenced by the effect of admixture in the populations; for example, the Algerian Berber population presents some individuals with high admixture with sub-Saharan Africa, which could provide an increase of the Ne due to recent events.

**Fig. 5.**
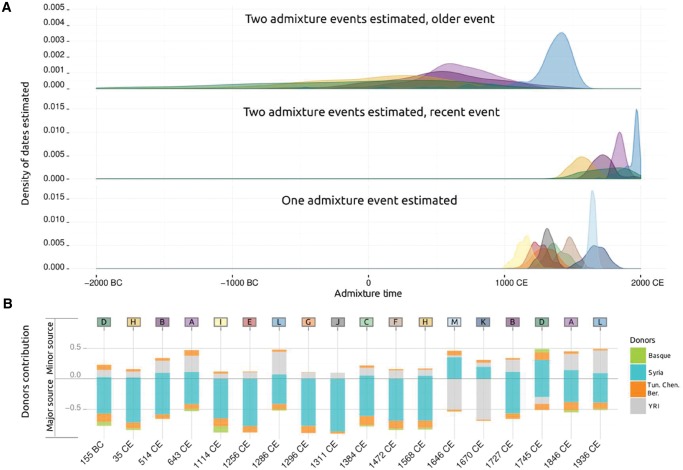
Globetrotter estimations in North Africa. (A) The three density plots on the top of the figure show the admixture times estimated for each cluster for each of 100 bootstrap re-samples: top, older event when two admixture events are estimated; middle, recent event when two admixture events are estimated, and; bottom, only one admixture event estimated. (B) The barplot at the bottom of the figure shows the proportion of the four components (Middle Eastern, North African, sub-Saharan, and European) inferred in each cluster, considering the minor source (up) and major source (down) and the estimated times of admixture.

We used GLOBETROTTER to identify and date admixture events, using the same four surrogate groups: Yoruba (YRI), as representative of sub-Saharan Africa; Basques, as European proxies; Syrians, as Middle Easterns; and Tunisian Berbers from Chenini, as North African donors. Results suggest a history of gene flow that can be dated to as long ago as the first century B.C. (fig. 5 and supplementary table S1, Supplementary Material online). For eight clusters, we detect a single pulse of admixture, whereas in five clusters we find evidence for multiple waves of admixture. In the latter scenario, with the exception of cluster D, we infer that the sources that intermixed at different times had a similar genetic make-up, which is consistent with both multiple pulses and continuous admixture over the inferred dates between two distinct source groups (fig. 5B). In most clusters, the minor contributing source is primarily represented by Yoruba and a variable proportion of Syria and North African-like ancestry, whereas the major contributing source is primarily composed of a Syria-like contribution and smaller contributions from North Africa and in some cases Basque. Overall the GLOBETROTTER results suggest complex admixture where recently admixing source populations were already admixed and/or where multiple different groups mixed at around the same time (see fig. 5, supplementary figs. S12 and S13, and table S1, Supplementary Material online), making interpretations challenging. Nonetheless, we detect two separate waves of admixture. Estimated dates of the oldest admixture wave are less precise than the recent one, as shown by their largest standard deviations (supplementary table S1, Supplementary Material online), although two coincident peaks around the 7th century are detected for both the A and B clusters (fig. 5A). The most recent wave of admixture starts around the 10th century and expands almost until the present. At least in three clusters (K, L, and M) there is substantial contribution of the sub-Saharan surrogate group (YRI). For cluster L two events are detected, one dated to around the 13th century and another dated to the 19th century, which is consistent with sub-Saharan continuous gene flow up to very recently. In contrarst, clusters K and M show coincident sub-Saharan migration dates around the 17th century.

## Discussion

Our genome-wide results for several human groups in North Africa, including several Berber groups, confirm their genetic heterogeneity and the complex demographic history of the area. This complexity highlights the high degree of inter-population admixture and the challenge of defining genetic groups in North Africa. Populations, understood as groups of individuals sampled together to be analyzed and described as an entity, are usually geographically determined considering the birth place of the individuals and their close ancestors. However, when these *sampled populations* are studied from a genetic point of view, genetic groups, or *clusters*, can be established based on genetic similarities rather than their geographical origin. Some individuals show high correspondence between their geographical origin and their genetic affinities, as shown for example in European populations (Lao et al. 2008; Novembre et al. 2008; Busby et al. 2015). Consistent with this, our analyses show that European, Middle Eastern, and sub-Saharan populations exhibit a homogeneous genetic structure in accordance with their geographical sampling. However, North African populations do not show this correlation, and individuals from the same geographical origin are distributed in different clusters, or genetic groups, with no clear demographic, ethnic or geographical classification (fig. 3).

The lack of correlation between geographic and genetic structure and the high heterogeneity shown within North African groups largely can be explained by heterogeneous or unbalanced admixture. Our results show that differential admixture patterns with other populations, mainly from Middle East and sub-Saharan Africa, and to a lesser extent Europeans, are added to any autochthonous genetic component in North African individuals. North African individuals with very similar admixture patterns tend to group despite their geographical origin, and thus the same fineSTRUCTURE cluster or genetic group can be found in different geographical populations due to similarities in the admixture patterns of the individuals (supplementary fig. S8, Supplementary Material online). These differential admixture patterns, in the apparent absence of strong levels of within-population drift following admixture, at least partially explain the high heterogeneity found within geographical groups of individuals sampled from the same location. This fact is especially remarkable in the Zenata Berber population from Algeria, where genetic similarity to sub-Saharan Africa in some individuals is extremely high, whereas similarity to the Middle East is much higher in others (fig. 3 and supplementary fig. S8, Supplementary Material online). Substructure within geographical populations may be caused by social substructure; for example, the sub-Saharan component is believed to come at least in some cases from recent slave trade (Harich et al. 2010), and social structure may influence which individuals are affected by the admixture in that case. Moreover, the heterogeneity and admixture patterns observed in North Africa suggest a high amount of migration within the region without a clear pattern. Nevertheless, some genetic patterns can be related to geography, such as the one shown by cluster H, which is a genetic group spread along the Mediterranean coast and might have been related to migrations along the coast (fig. 3).

North African populations are also ethnically complex, and it is common to differentiate between Arab and Berber (Amazigh) groups based on cultural practices, such as language. Although historically and sociologically this consideration is assumed, no genetic differences have been reported between Arabs and Berbers when analyzing individual genetic markers (Bosch et al. 1997, 2001; Plaza et al. 2003; Arredi et al. 2004; Coudray et al. 2009; Fadhlaoui-Zid et al. 2011b; Fadhlaoui-Zid et al. 2013; Bekada et al. 2015). A previous genome-wide analysis performed with ∼900 K autosomal markers (Henn et al. 2012) showed a distinction of a single Berber sample from Tunisia (from Chenini) compared with several Arab populations, with those Berbers presenting the higher frequency of the presumed North African autochthonous (aka Maghrebi) genetic component. However, the present analysis of additional Berber samples reinforces the idea of no strong genetic distinction between Arabs and most Berber groups. Our results show that Berber groups, similar to the rest of North African populations, are very heterogeneous and have experienced a history of high admixture and contact with other populations that, if ever existed, have dissolved their common genetic background. Two Berber-speaking Tunisian samples analyzed, Chenini and Sened, show a genetic homogeneous pattern, such that nearly all individuals from each are assigned to clusters containing only individuals with the same label (fig. 3) and higher evidence of interbreeding signals that might be explained by their geographical isolation and limited contact with other populations. However, other Berber-speaking groups, such as the two Moroccan Berbers analyzed, are genetically heterogeneous and diverse, which might be explained by continuous contacts and admixture with neighboring populations. Finally, the Zenata Berber sample from Algeria shows a high degree of admixture with sub-Saharan Africa in recent times (figs. 3 and 5). In sum, our results show that there are many differences in the genetic structure of Berbers depending, at least, on their recent history, and thus not all Berber groups might be considered genetically isolated or homogeneous. Despite this, the Mozabite, a group of Algerian Berbers, are usually considered a genetic representative population of North Africa and even the Middle East (taking the term MENA, Middle East/North Africa), because it is the only North African population present in reference panels (such as the HGDP; Cann et al. 2002), even though some sub-Saharan admixture has been detected (Hellenthal et al. 2014). Uniparental markers have shown that there is high heterogeneity within Algerian groups despite their ethnic affiliation (Bekada et al. 2015), and our genome-wide analysis highlights the genetic complexity of North African groups and challenges the use of a single North African sample, such as the Mozabite, as the proxy for the North African genetic diversity.

The estimation of the dates of admixture in North African populations is not an easy task, as a large number of potential ancestry components (sub-Saharan, Middle Eastern, and European), some of which have likely diverged from one another relatively recently, are difficult to differentiate. We have addressed this issue by the use of haplotype-based methods that can have more precision to detect signals of historical and recent admixture events (Hellenthal et al. 2014). Our data show that contacts with diverse populations in North Africa have been continuous at least during recent history, which implies that substantial admixture between different groups might have taken place slightly before the beginning of the current era. The admixture events estimated in North African around 7th century C.E. (fig. 5) are in agreement with the Arabic expansion in the region. A complex pattern of contributing sources is shown, with a main Middle Eastern contribution in all samples, but also a sub-Saharan contribution, which could have been introduced by the Arabs through the slave trade (Newman 1995). Moreover, the Arabic expansion is expected to produce significant changes both in the social and genetic structure of North Africa, producing not only gene flow from Middle East but also introducing a complex pattern of admixture of multiple sources, as is shown in these analyses. The present results suggest that some Berber groups, those less geographically isolated, might have incorporated Arab newcomers, although this introgression might have been different in Berber groups, which explains the genetic heterogeneity seen nowadays in Berbers. The incorporation of these Arab newcomers might have also induced a language replacement (from Berber to Arabic) in some groups, which would explain the lack of genetic differentiation observed in our results between Arab- and Berber-speaking groups. Therefore, our results show that the Arabization, the expansion of the Arab culture and language from the Arabic Peninsula to the Maghreb (i.e., Northwest Africa) starting in the 7th century C.E., was mainly a demographic process that implied gene flow and remodeled the genetic structure, rather than a mere cultural replacement as suggested previously by historical records (McEvedy 1995; Newman 1995) and uniparental markers (Bosch et al. 2001; Arredi et al. 2004). Our most recent estimated dates correlate with sub-Saharan admixture in North Africa, which is continuous during the last few centuries (from the 13th century to the 20th century, see cluster L in fig. 5), as previously suggested by historical records (Newman 1995) and genetic data (Harich et al. 2010; Henn et al. 2012). However, it is noteworthy that very precise dates are found in some cases in the 17th century in western clusters (see cluster K and M). The admixture dates in the 17th century could be the consequence of the trans-Saharan slave trade that resulted from the Ottoman rule in North Africa and the arrival of the Crown of Castile and the Portuguese Kingdom to the West African seaports in the 16th century. The Iberian presence, driven by the search of a workforce in their recent settled Atlantic territories, modified the political and socioeconomic structure of Western Africa. This also intensified traffic through trans-Saharan routes to North Africa after the emergence of the sugar industry in this region and the Atlantic territories (Newman 1995; Oliver and Atmore 2001; Da Mosto 2003). Comparison of inferred ancestry proportions between the autosomes and X chromosome in Cluster M is indicative of sex-biased admixture with an overabundance of males with Middle Eastern (Syrian-like) ancestry and females with sub-Saharan African (Yoruba-like) ancestry.

Moreover, we infer a lower proportion of sub-Saharan ancestry older than previously described in all admixture events dated from the first century B.C., which could be attributed to more ancient slave trade during the Roman or Islamic periods, such as the servile Haratin population of Nilo-Saharan origin in Berber groups such as the Sanhadja and Zenata (Newman 1995). Caution is warranted, however, as there are serious difficulties in reliably estimating the proportions contributed by each source population in the admixture events, mainly because the lack of a proper ancestral North African population. In our analyses, we have considered the population from Tunisia Chenini as the best proxy, but genetic drift in Chenini samples due to isolation and interbreeding might substantially underestimate the contribution of the autochthonous ancestral groups in extant North African populations.

The recent genetic heterogeneity of North African groups described in the present analysis highlights the pivotal need of taking into account genetic substructure in future GWAS applications and other analyses of complex traits when North African samples are considered. The inclusion of cases and controls without accounting for substructure or the simple distinction of Arab and Berber groups (see for instance, Ross et al. 2011) might be inadequate and lead to spurious results due to the strong genetic heterogeneity. Special caution should be taken when matching cases and controls in North African GWAS since differences in admixture components in cases and controls might led to false genetic associations. The increase of population genetic analyses of North African groups, including more Berber-speaking samples, might refine our knowledge of the heterogeneity found in the region. Even if genetic differences seem to have originated in recent times, the study of more Berber groups and finer analyses, like whole genome sequencing and the presence of ancient DNA samples in the databases, would allow us to go in depth into their ancient history and trace their origins.

## Materials and methods

### Samples and Genotypes

New Berber samples were genotyped in the present study, which include samples from two populations from Morocco: Tiznit and Errachidia; Zenata Berbers from Timimoum in Algeria; and Tunisians from Sened. A Syrian sample was also genotyped (supplementary table S2, Supplementary Material online). The sample set was formed by self-reported non-related volunteers with the corresponding informed consent. Written informed consent was obtained from the participants and analyses were performed anonymously. The present project obtained the ethics approval from the Institutional Review Board, *Comitè Ètic d’Investigació Clínica-Institut Municipal d’Assistència Sanitària* (CEIC-IMAS) in Spain (2013/5429/I), as well as the approval from the local committees of the Charles Nicolle Hospital in Tunis, Tunisia; the CRASC (*Centre de Recherche en Anthropologie Sociale et Culturelle*) in Oran, Algeria; and the *Comité d’Éthique du CHU (Centre-Hospitalo-Universitaire) Mohamed VI* in Marrakech, Morocco. DNA was extracted from blood samples using standard protocols, all samples were genotyped with Affymetrix 6.0 array and genotype calling was performed using the Affymetrix genotyping console 4.1.3.840 (data available in https://figshare.com/articles/North_African_Berber_dataset/3501761). The genotype calling was performed with the present data set and data from HapMap (The International HapMap Consortium 2003), Henn et al. (2012) and Botigué et al. (2013) in order to increase genotyping accuracy. SNPs missing in more than 90% of the individuals, those that failed Hardy–Weinberg test at 0.05 significance threshold, and those with a minor allele frequency (MAF) below 0.05 were discarded. Individuals sharing more than 85% of their genome identity by state (IBS) were removed, and remaining individuals with more than 90% of missing SNPs were also excluded. For the analyses that required linkage equilibrium between SNPs, SNPs were pruned using a pairwise linkage disequilibrium maximum threshold of 0.5 (for the X chromosome the coefficient threshold was set to 0.8 in order to increase the number of SNPs) using a windows size of 50 a shift step of 5. The same quality control filters were applied for the X chromosome and the autosome markers separately using PLINK 1.07 (Purcell et al. 2007).

The newly genotyped samples were merged with published data from Middle East, sub-Saharan Africa, Europe, and North Africa. Populations from the HGDP (Cann et al. 2002), HapMap (The International HapMap Consortium 2003), Lebanon (Haber et al. 2013), and Qatar (Hunter-Zinck et al. 2010) were combined in different datasets that differ in SNP density. Then, depending on the SNP density required for each analysis the appropriate dataset was used. Individuals from the reference populations were normalized to a maximum of 25 individuals per population whenever possible in order to avoid biases due to different sample sizes (supplementary tables S2 and S3).

### Population Structure Analyses

Principal component analyses (PCA) were performed using the SmartPCA program from the EIGENSTRAT stratification correction method implemented in EIGENSOFT 4.2 package (Patterson et al. 2006). Fst values between each population were also estimated using EIGENSOFT 4.2. ADMIXTURE (Alexander et al. 2009) was run to explore patterns of population structure, testing from *k* = 2 to 10 ancestral clusters using 10 different random seeds. Plots were represented using the software Distruct1.1 (Rosenberg 2004).

### Interbreeding and Effective Population Size (Ne) Analyses

Runs of homozygosity (ROH) analyses were performed in order to test for inbreeding among North African populations (excluding Mozabites from HGDP to increase the number of SNPs), using SNPs at all allele frequencies and allowing for linkage disequilibrium between them, leaving a total of 200,538 SNPs. Runs were identified in PLINK using a window of 5,000 kb and sliding it across the genome allowing for one heterozygous and one missing call per window. The minimum length of each ROH was set to 500 kb with 25 SNPs and a maximum gap of 100 kb between two consecutive SNPs.

To estimate the effective population size (Ne) of each population we followed an approach similar to Li et al. (2008). LDhat (Auton and McVean 2007) was used to estimate population-based recombination rates on all autosomes using a sliding window of 2000 SNPs with an overlap of 500 SNPs between contiguous windows. The number of iterations was set to 10^7^, the thinning interval to 2000 and the burn-in value to 500 (discarding 10% of all retained observations). The penalty for a change in recombination rate was set to 20. The number of individuals on each population was normalized to 13. The effective population size *N*_e_ was then estimated using the slope of a simple linear regression of population-based (*ρ*) on pedigree-based, sex averaged, deCODE recombination rates (*r*) (Kong et al. 2010) without the intercept.

### ChromoPainter and Globetrotter Analyses

We used SHAPEIT (O’Connell et al. 2014) to phase the data, using the population-averaged genetic map from the HapMap phase II (The International HapMap Consortium 2003) and the 1000 genomes dataset as a reference panel (The 1000 Genomes Project Consortium 2012). This phasing step was performed after an alignment with the reference panel and the removal of SNPs that did not align. Population structure was studied using ChromoPainter v2 (Lawson et al. 2012). Briefly, ChromoPainter composes the two haploid genomes of a “recipient” individual as a mosaic of the haploid genomes of a set of “donor” individuals, inferring which donor the recipient is most closely related to (out of all donors) at each genetic location along the recipient’s genome. In this manner, ChromoPainter infers the total number and length of haplotype segments for which the recipient shares a most recent common ancestor with each donor.

When running ChromoPainter, we treated separately each sampled individual in our dataset as our recipient, painting this recipient using all other sampled individuals as donors. We first inferred the global mutation probability and the switch rate for chromosomes 1, 7, 14, and 20 in 10 iterations of the EM (expectation maximization) algorithm, the obtained values were: 0.00037 and 340.06914, respectively. We then fixed these parameters to infer the final ChromoPainter coancestry matrix that measures the amount of haplotype sharing among individuals summed across all chromosomes.

FineSTRUCTURE (Lawson et al. 2012) was used to cluster individuals into genetically homogeneous groups based on this coancestry matrix, using the number of chunks copied among individuals (chunkcounts). Following Leslie et al. (2015), we ran fineSTRUCTURE for 2 million Markov-Chain-Monte-Carlo (MCMC) iterations, discarding the first 1 million iterations as “burn-in”, sampling from the posterior distribution every 10,000 iterations following “burn-in”. Following the standard fineSTRUCTURE protocol, we then found the MCMC iteration with the highest posterior probability, and performed 10,000 “hill-climbing” iterations as described in (Lawson et al. 2012) to get final cluster assignments. We then built a tree relating these final clusters by pairwise merging similar clusters in a greedy fashion (Lawson et al. 2012). Heatmaps of the coancestry matrices (using each the total number and total length—in centimorgans— of shared haplotype segments), with individuals ordered according to the fineSTRUCTURE tree, are shown in supplementary figures S6 and S7, Supplementary Material online. We then classified individuals into final groups labeled as clusters A–M (fig. 2) based on the branches of the fineSTRUCTURE tree (first tree in supplementary fig. S11, Supplementary Material online) and visual inspection of the coancestry matrix in order to identify groups of genetically homogeneous individuals. Thus, North African populations were classified in clusters from A to M, along with separate clusters for Basques, Syrians, YRI, Tunisian Berbers from Chenini and CEU, each of which formed homogeneous clusters according to fineSTRUCTURE. One individual from Tunisia Sened and four YRI were removed because of relatedness or because they were visual outliers in the ChromoPainter results. We repeated our fineSTRUCTURE clustering and tree inference three times using different seeds (supplementary fig. S11, Supplementary Material online), though results were similar for all three, sometimes simply moving a couple of individuals into genetically similar clusters nearby in the tree.

We ran GLOBETROTTER separately on each of our North African clusters A-M to test for instantaneous episodes of admixture. For this analysis we ran again ChromoPainter v2 in a separate analysis using the same switch rate and global mutation parameters cited above, but individuals were a priori classified into groups based on fineSTRUCTURE clustering results. Syrians (representing the Middle East), Basques (representing Europe), YRI (representing sub-Sahran Africa), and North African cluster from Tunisia Chenini were considered as both donors and recipients, whereas the rest of North African groups A–M were considered as recipients.

In order to check the accuracy of the reference source populations chosen for sub-Saharan Africa and Europe we ran again the ChromoPainter analyses with other available populations of the area keeping all the parameters as previously. We included GBR, TSI, and IBS populations from 1000 Genomes Phase 3 (The 1000 Genomes Project Consortium 2012) to use as European source populations together with Basques and CEU from previous analyses. We also use MSL and LWK from 1000 Genomes Phase 3 as sub-Saharan source populations together with the Yoruba used in the main analysis (supplementary fig. S14). No Middle Eastern samples with a similar SNP dataset for comparison to our samples were available besides our own genotyped Syrians, and therefore only this sample is used as a Middle Eastern proxy. In the same way, the cluster from Tunisia Chenini was chosen as an ancestral proxy for North Africans due to the genetic homogeneity and little admixture of this sample as shown in (Henn et al. 2012). We ran GLOBETROTTER (Hellenthal et al. 2014) as previously described (van Dorp et al. 2015), testing each North African cluster separately and considering the four donor groups cited above as surrogates. We used ten painting samples per individual and the coancestry matrix for the total genome-wide length of haplotype sharing obtained from ChromoPainter. For all results presented here, when analyzing each North African cluster we standardized each coancestry curve by a “NULL” individual designed to eliminate any spurious linkage disequilibrium patterns not attributable to that expected under a genuine admixture event (Hellenthal et al. 2014), though we note results were similar when not performing this standardization. We ran 100 bootstrap iterations for estimating admixture dates, a time generation of 28 years was considered for all the analyses. The proportions of haplotype sharing between each target individual and the four surrogate groups was inferred performing a non-negative-least squares (NNLS) on each recipient individual as described in (Leslie et al. 2015; Montinaro et al. 2015), i.e., using the inferred proportions of haplotype sharing for the recipient individual as a response and the inferred proportions of haplotype sharing for the four surrogate groups as predictors.

### Comparison of Ancestry Estimates on the X Chromosome and Autosomes

In order to test for sex-biased gene flow, we phased the X chromosome using the same parameters as for the autosomes (see above); the phasing output by default treats each male individual as double homozygous, which is convenient when running the same analysis as autosomes. We then ran ChromoPainter on each individual sample for the X using the same parameters, donors and recipient clusters as for the autosomes (see above). The average ancestry proportions that each North African recipient group shares with the four surrogate groups were inferred by performing a NNLS on the X chromosome haplotype sharing results analogous to the autosomes (see above). Finally, to test for sex-bias gene flow, we subtracted the X and autosomal contributions from each surrogate for each North African recipient cluster. To evaluate the robustness of this difference, we performed bootstrap analysis for each North African recipient cluster separately. About 10,000 independent bootstrap draws were performed. For each bootstrap draw, *n* individuals were sampled with replacement, where *n* is the size of the North African cluster. The proportions of haplotype sharing for these resampled individuals were averaged to produce the average proportion of haplotype sharing for this resampled North African cluster, which was then used in NNLS to obtain the ancestry proportions for this resampled cluster. After estimating the X chromosome and autosomal ancestry proportions for the resampled cluster (same set of resampled individuals used for both) the differences between the X chromosome and autosomes were recorded. Therefore, for each specific North African recipient cluster and each donor group, we had 10,000 bootstrap values that provided the bootstrap distribution of the observed X chromosome vs. autosomal ancestry difference. To estimate significance, we counted how many times this difference was greater (or smaller) than 0 (i.e., no difference in ancestry proportions of the X chromosome vs. autosomes) out of 10,000 bootstraps, and this proportion was multiplied by 2 to get a two sided *P*-value. With 13 recipient and four donor groups, a Bonferroni-adjusted threshold of 0.05/52 = 0.00096 was used for significance. We analyzed chromosome X vs. only chromosome 2, to have a comparison where both sides had roughly equal number of SNPs (27,315 SNPs for X chromosome and 24,867 SNPs for chromosome 2); it gave quite similar results to the X chromosome vs. whole autosome analysis (supplementary fig. S9, Supplementary Material online), the *P*-values being slightly weaker due to the lower number of SNPs used. We also compared inferred ancestry proportions for chromosomes 1–6 to those using the rest of the autosomes, separately (supplementary table S4, Supplementary Material online). For the main discussion, we focus on the results comparing X chromosome against the whole autosomes as that contains the most SNPs.

## Supplementary Material

Supplementary figures S1–S14 and tables S1–S4 are available at *Molecular Biology and Evolution* online.

## Supplementary Material

Supplementary Data
