# Supplementary material for: Recent Historical Migrations Have Shaped the Gene Pool of Arabs and Berbers in North Africa
Source: Mol Biol Evol. 2016 Oct 15;34(2):318–29. doi: 10.1093/molbev/msw218 (PMC5644363; doi:10.1093/molbev/msw218)
Supplement: Supplementary Data [file msw218_Supp.pdf]

Supplementary Information

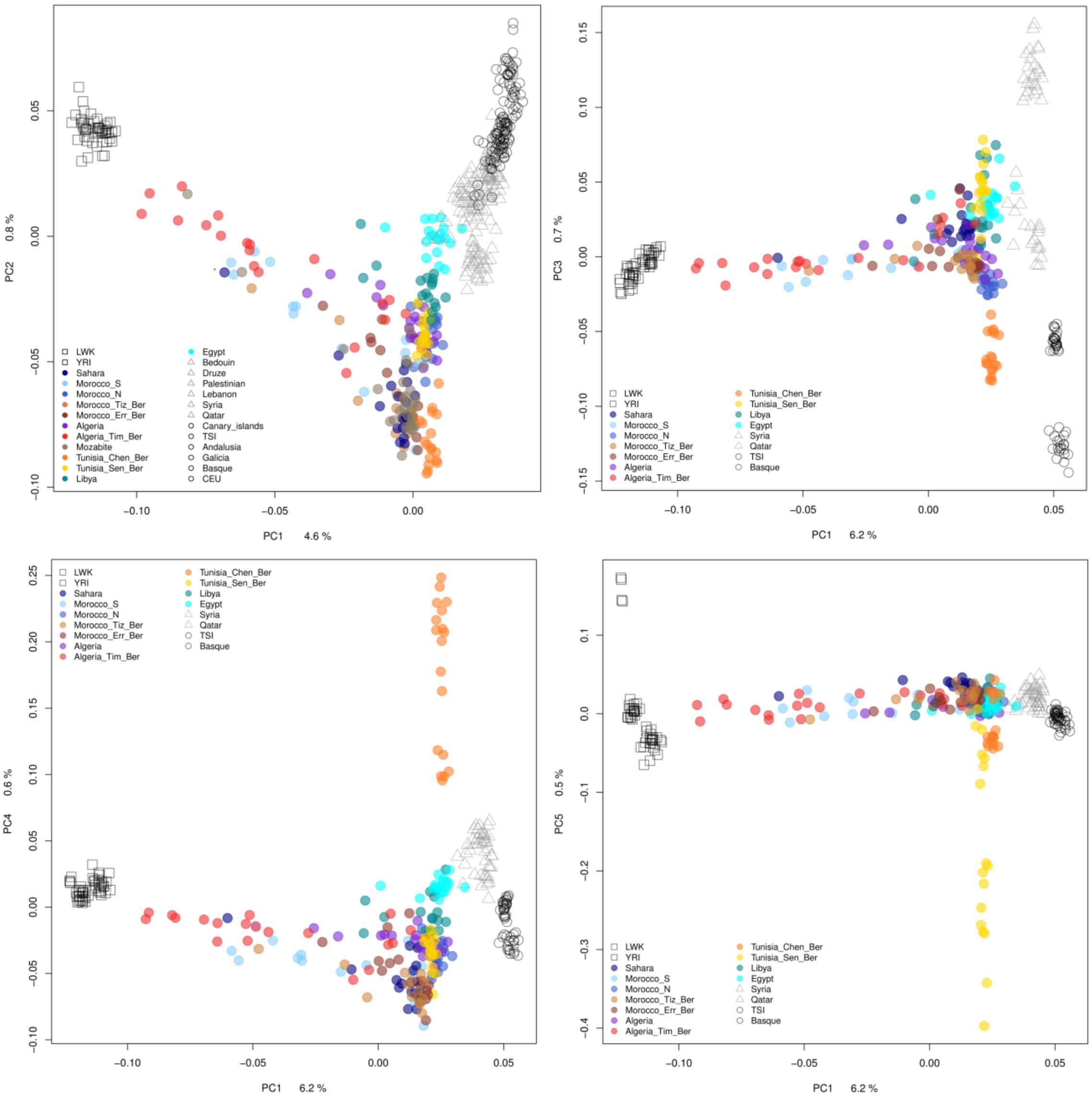

S1 Fig.Population structure. Principal Component Analysis. PC1 and PC2 including an extensive dataset of Middle Eastern and European populations (Table S1). PC1 vs PC3, PC4 and PC5 estimated with a reduced dataset of populations (the same dataset as in Figure 1).

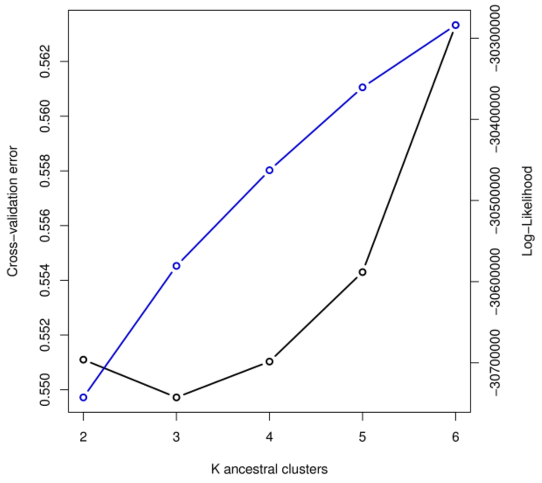

S2 Fig. **ADMIXTURE validation.** Cross-validation error and log-likelihood ratio for ADMIXTURE analysis in Figure 1.

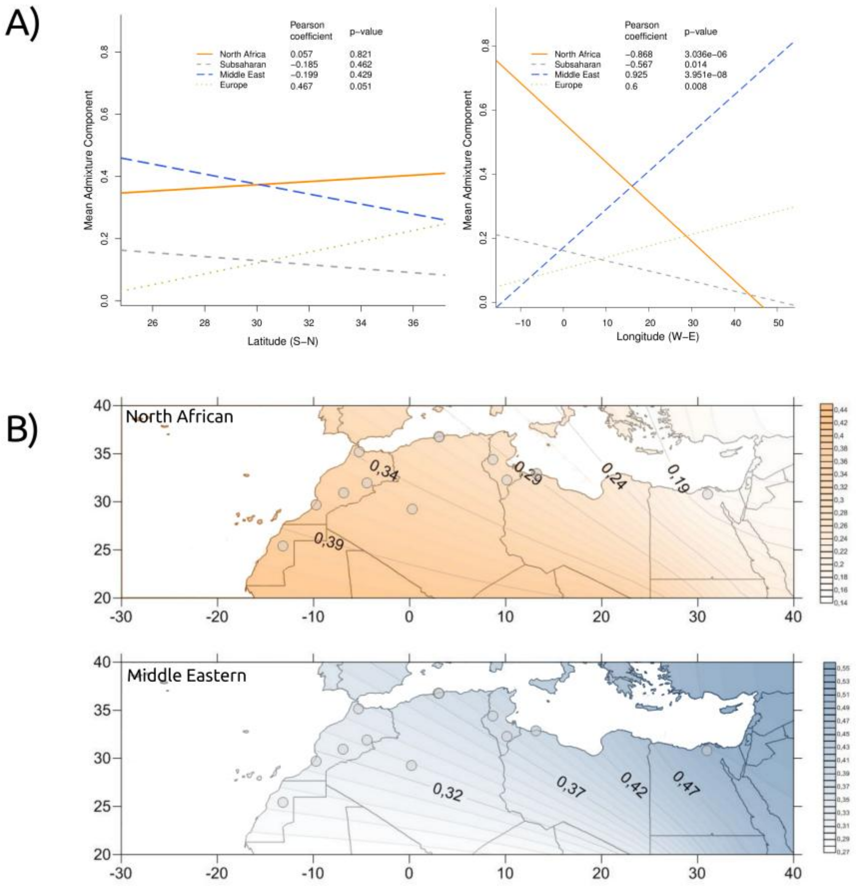

S3 Fig. Geographic correlation of ADMIXTURE. A) Correlation of geographical coordinates (Latitude and Longitude) with the mean of each component proportion given by ADMIXTURE in the North African dataset. B) Gradients of North African and Middle Eastern admixture components are shown in the maps.

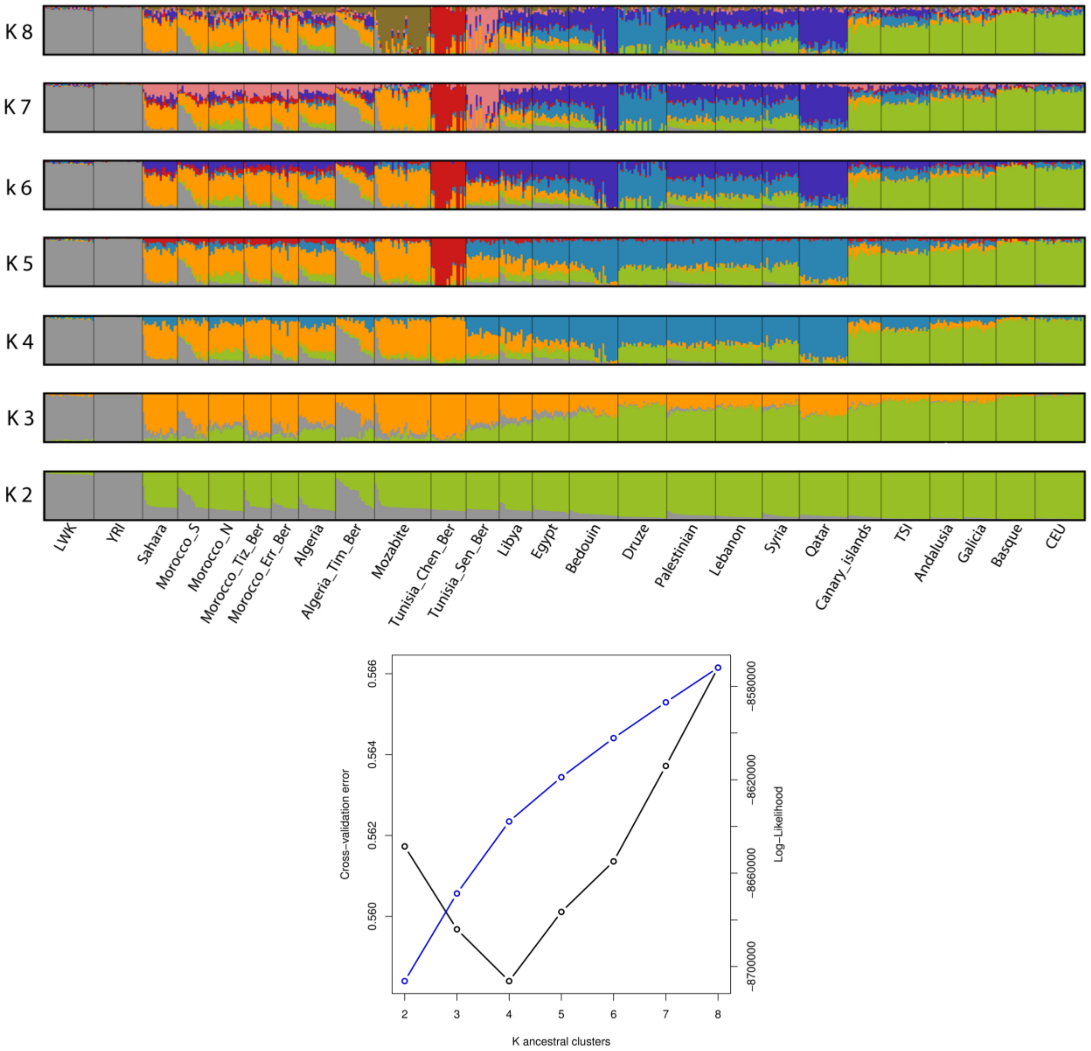

S4 Fig. **Population structure.** ADMXITURE plot and cross-validation and log-likelihood plot for a large dataset (Table S1) of populations from different array platforms and lower SNP density.

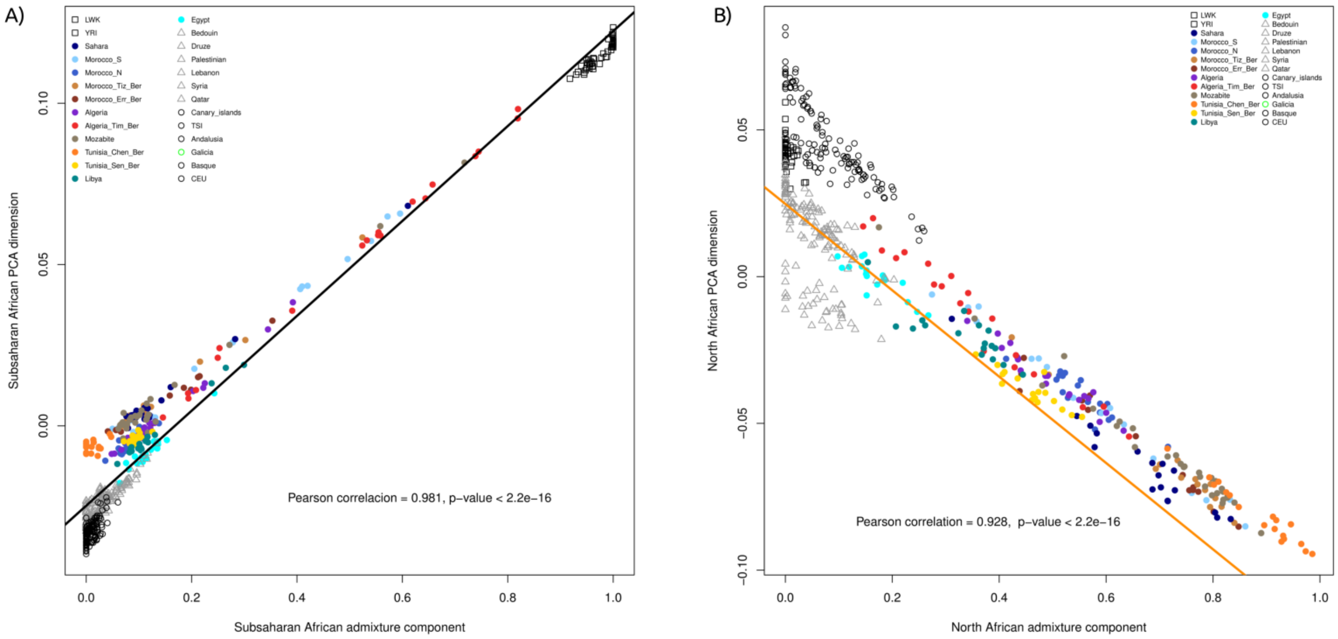

S5 Fig.PCA and ADMIXTURE correlation. Correlation between A) ADMIXTURE sub-Saharan African component and PC1; and B) North African ADMIXTURE component and PC2.

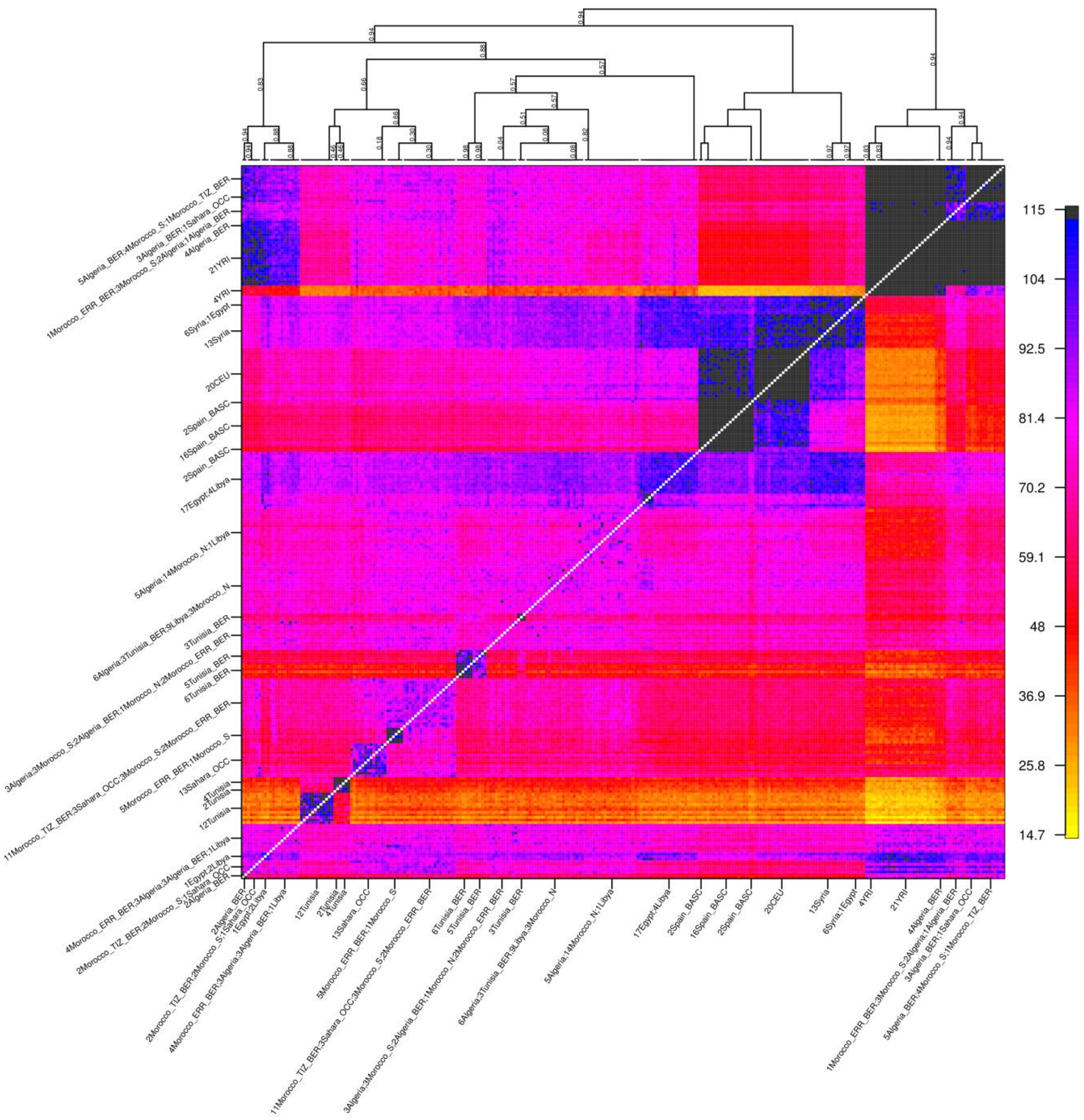

S6 Fig. Chunklength coancestry matrix. Heatmap and dendogram of the chunklenght coancestry matrix estimated with ChromoPainter setting all individuals as donors and recipients.

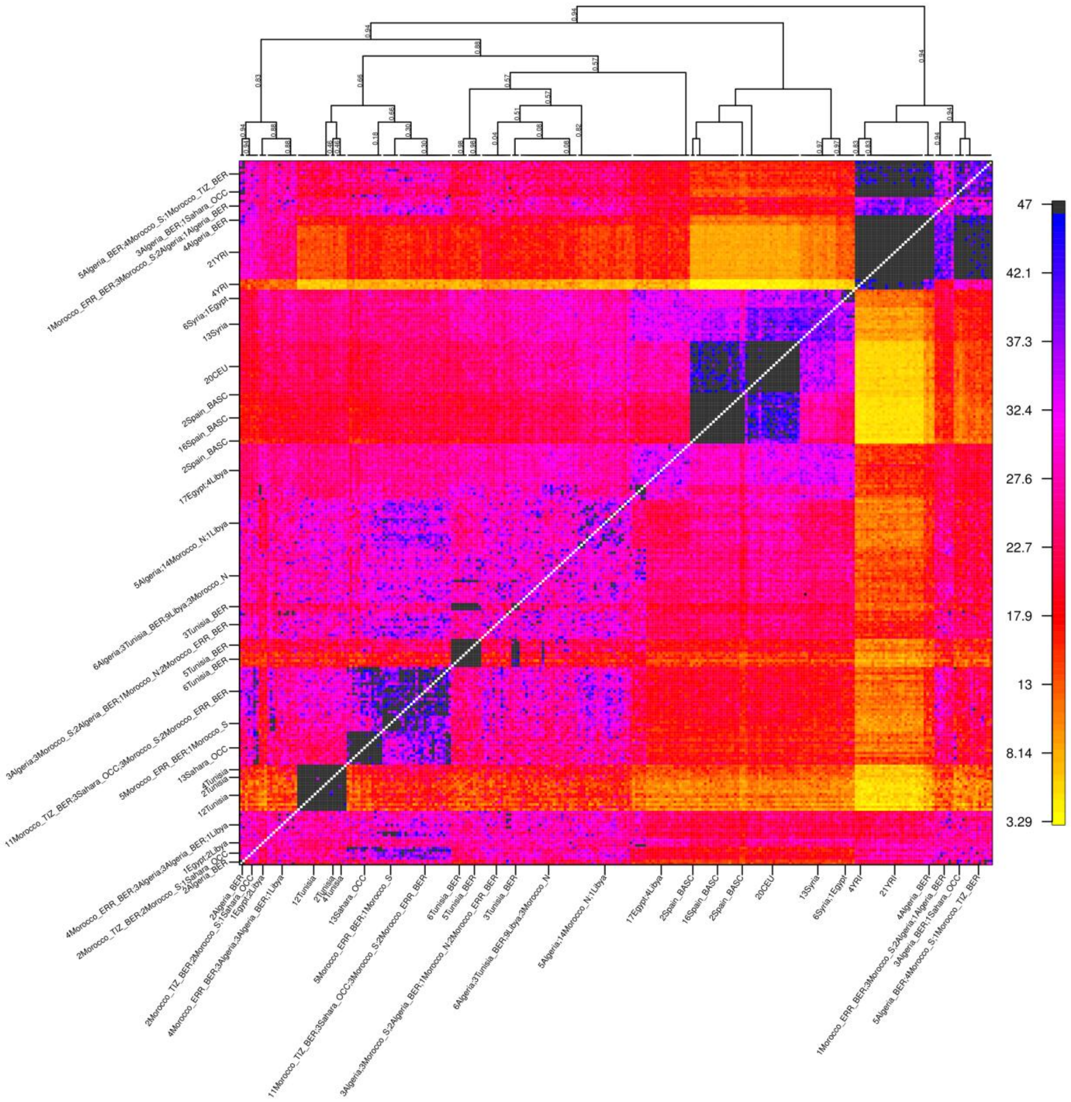
47
42.1
37.3
32.4
27.6
22.7
17.9
13
8.14
3.29

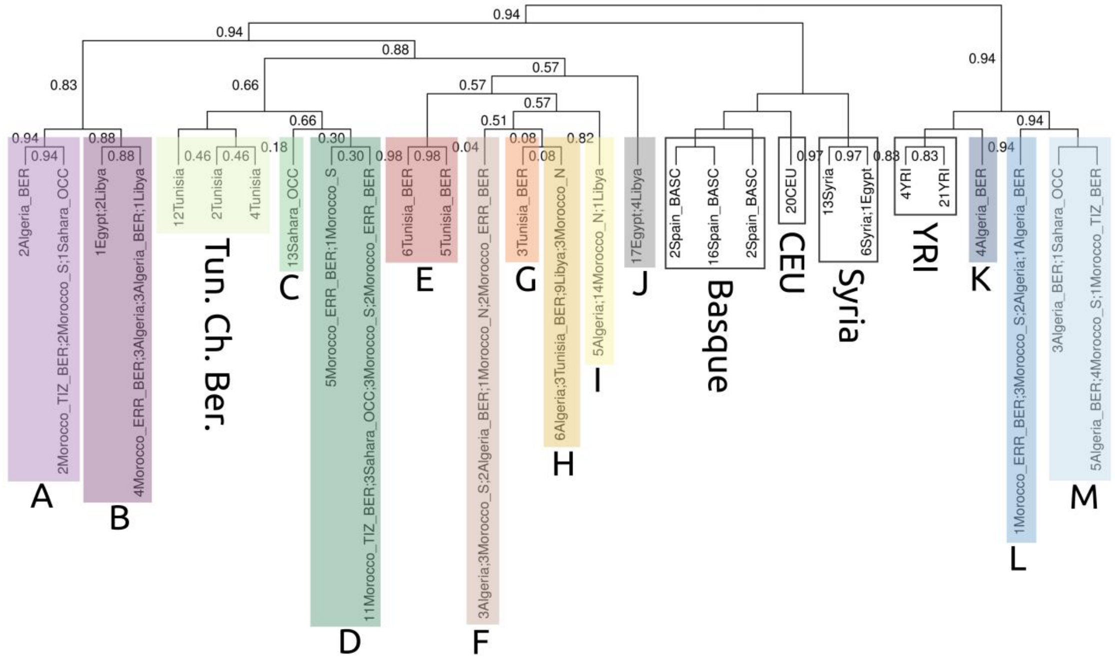
0.94
0.94
0.88
0.83
0.66
0.57
0.57
0.57
0.51
0.94
2Algeria_BER
2Morocco_TIZ_BER;2Morocco_S;1Sahara_OCC
1Egypt;2Libya
4Morocco_ERR_BER;3Algeria;3Algeria_BER;1Libya
12Tunisia
2Tunisia
4Tunisia
13Sahara_OCC
5Morocco_ERR_BER;1Morocco_S
11Morocco_TIZ_BER;3Sahara_OCC;3Morocco_S;2Morocco_ERR_BER
6Tunisia_BER
5Tunisia_BER
3Algeria;3Morocco_S;2Algeria_BER;1Morocco_N;2Morocco_ERR_BER
3Tunisia_BER
6Algeria;3Tunisia_BER;9Libya;3Morocco_N
5Algeria;14Morocco_N;1Libya
17Egypt;4Libya
2Spain_BASC
16Spain_BASC
2Spain_BASC
20CEU
13Syria
6Syria;1Egypt
4YRI
21YRI
4Algeria_BER
1Morocco_ERR_BER;3Morocco_S;2Algeria;1Algeria_BER
3Algeria_BER;1Sahara_OCC
5Algeria_BER;4Morocco_S;1Morocco_TIZ_BER
A
B
Tun. Ch. Ber.
C
D
E
F
G
H
I
J
Basque
CEU
Syria
YRI
K
L
M

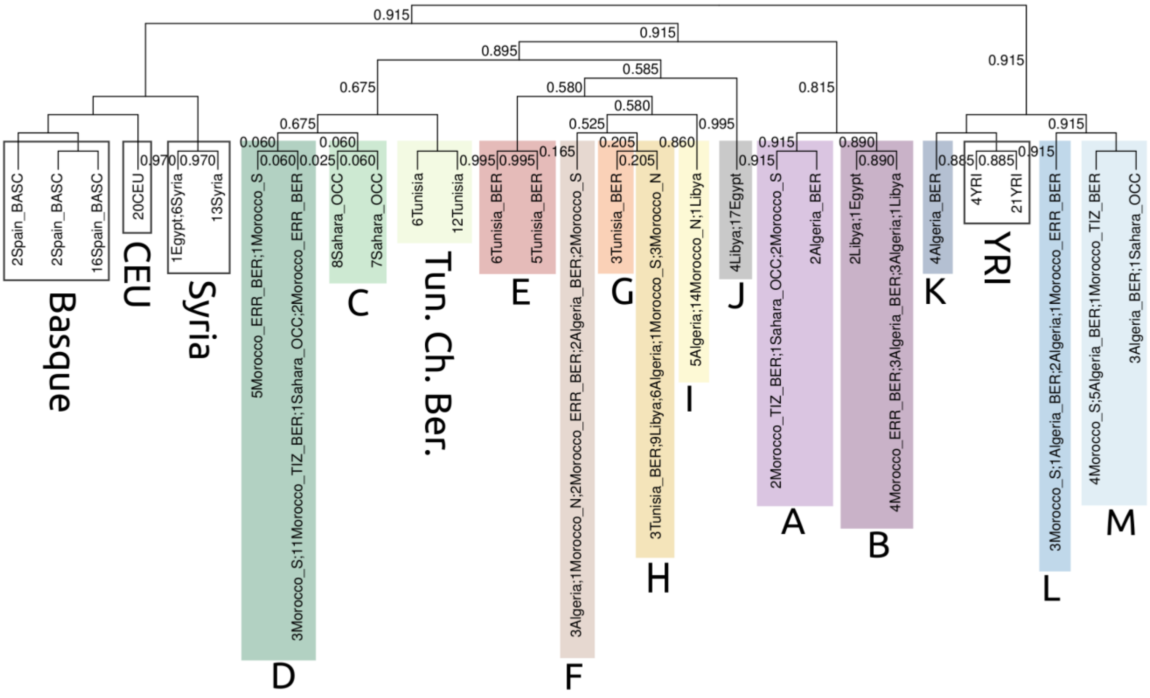

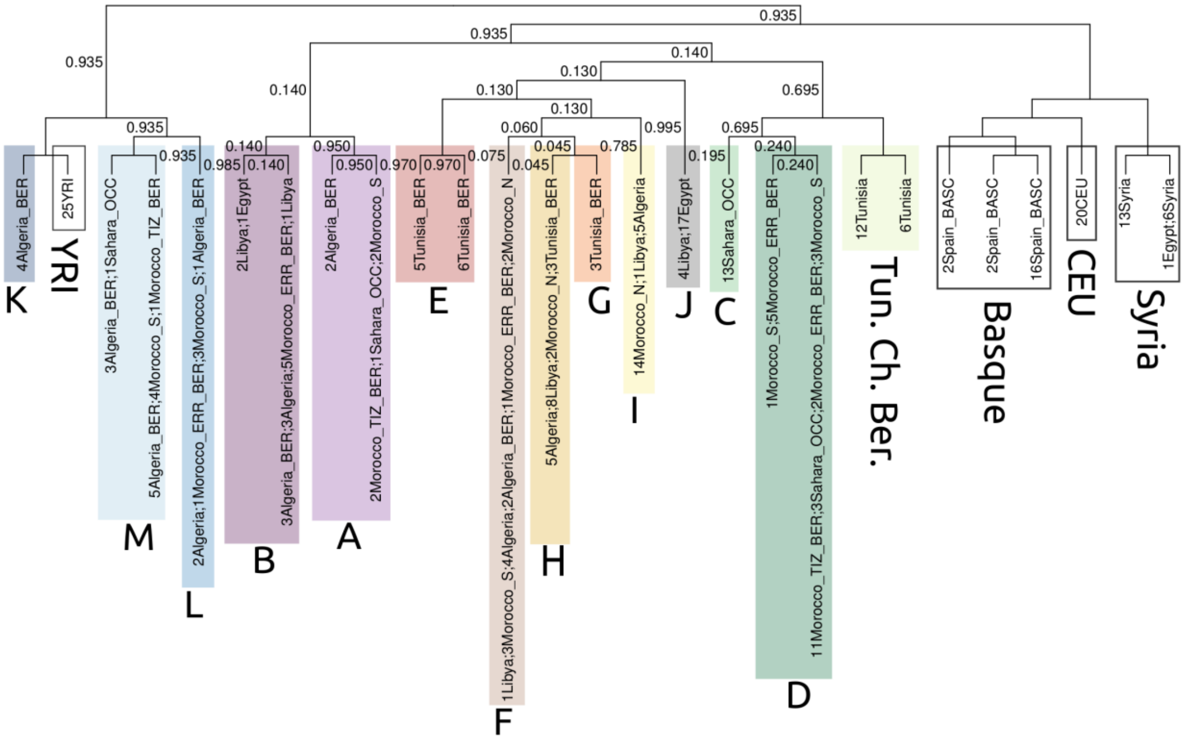

S7 Fig. Chunkcounts coancestry matrix and dendrograms. Heatmap and dendograms of the chunkcounts coancestry matrix estimated with ChromoPainter setting all individuals as donors and recipients. Three dendograms of FineStructure are shown for three different seeds.

YRI

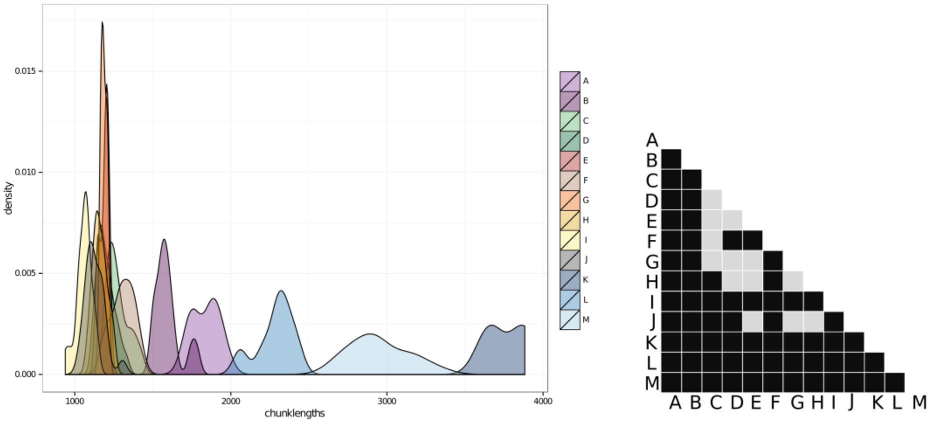

Basque

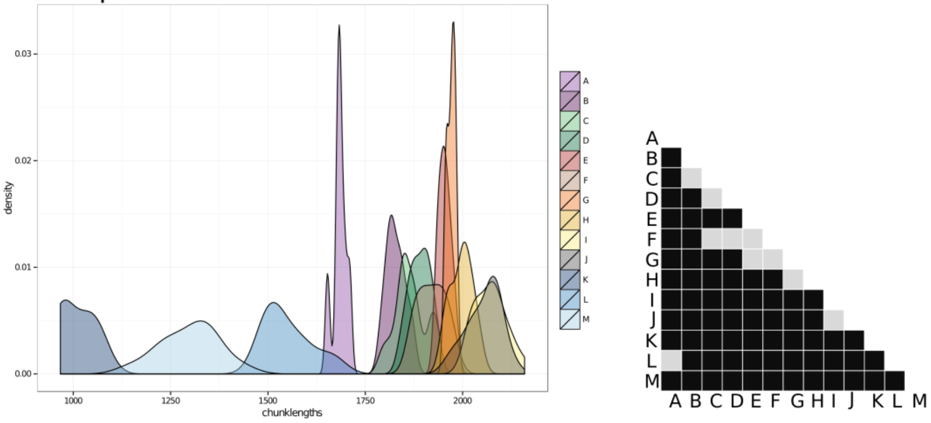

Syria

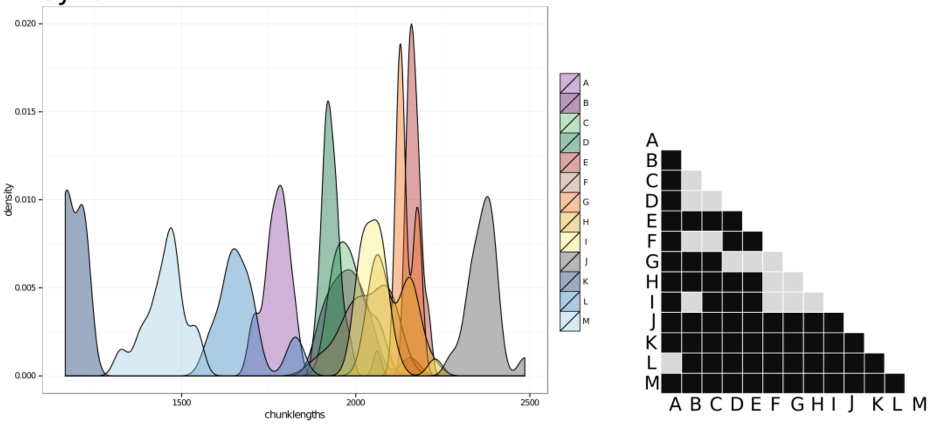

Tun_Chen_Ber

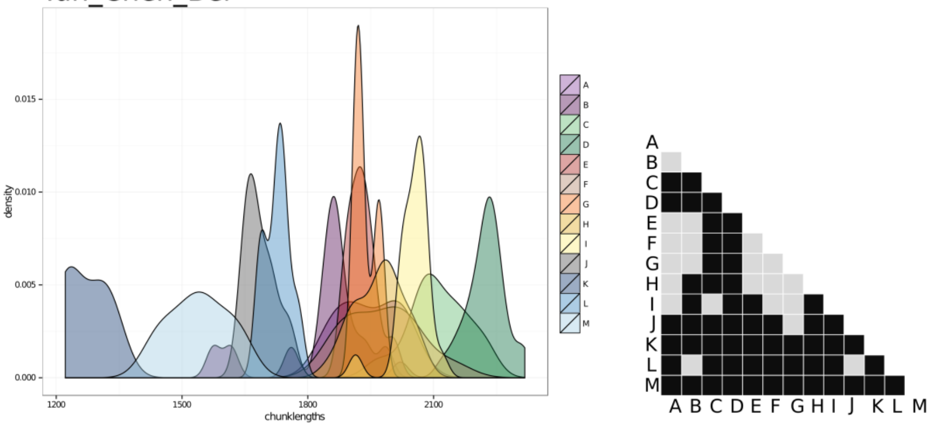

S8 Fig. Differential admixture. At the left,density plots for the total lengths of the chunks copied from each of the surrogate populations (YRI, Basque, Syria, Tun_Chen_Ber) to the individuals of each cluster (A-M) for the ChromoPainter analysissetting donors. At the right, significantly different (Bonferroni corrected p-values>0.05) pairs of clusters distributions compared with t-test are highlighted in the darker gray while non-significant values are painted in light gray.

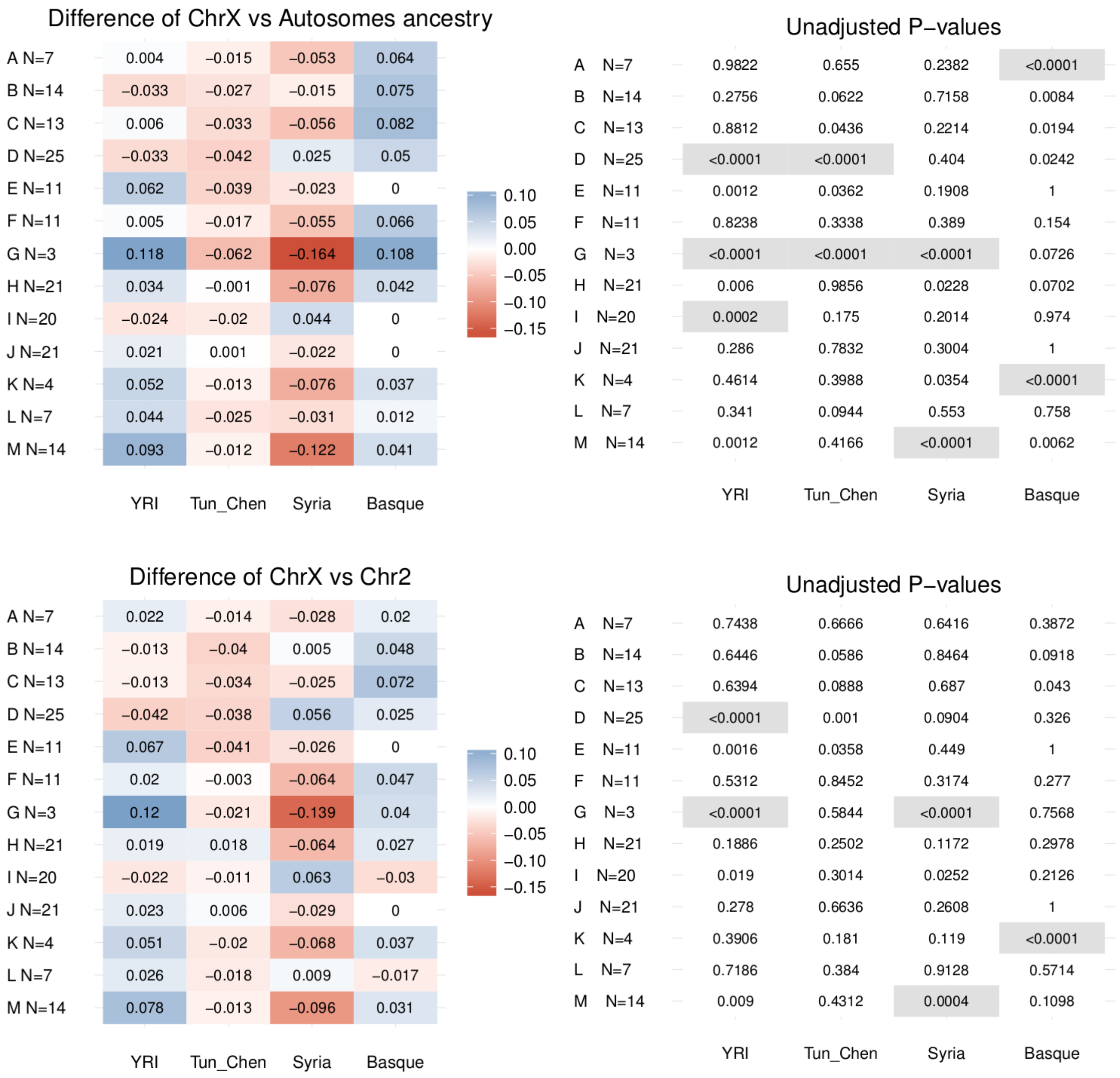

S9 Fig. Comparison of ancestry estimations on the X chromosome and autosomes. In order to detect sexual bias in the North African clusters, difference between the X and autosomals, X and chromosome 2 proportions of each ancestry for each North African cluster were estimated as described in the Material and Methods section. The comparison with chromosome 2 was chosen due to similar number and density of SNPs when comparing with the X chromosome. The left figures show the difference between the ancestry proportions of the four donors for the X chromosome and the autosomes and chromosome 2 and X, from top bottom. Positive values (blueish colors) indicate more ancestry proportion in the X chromosome, whereas negative values (reddish colors) indicate more ancestry proportion in autosomes or chromosome 2. P-values are shown on the right, highlighting the significant values after Bonferroni correction.

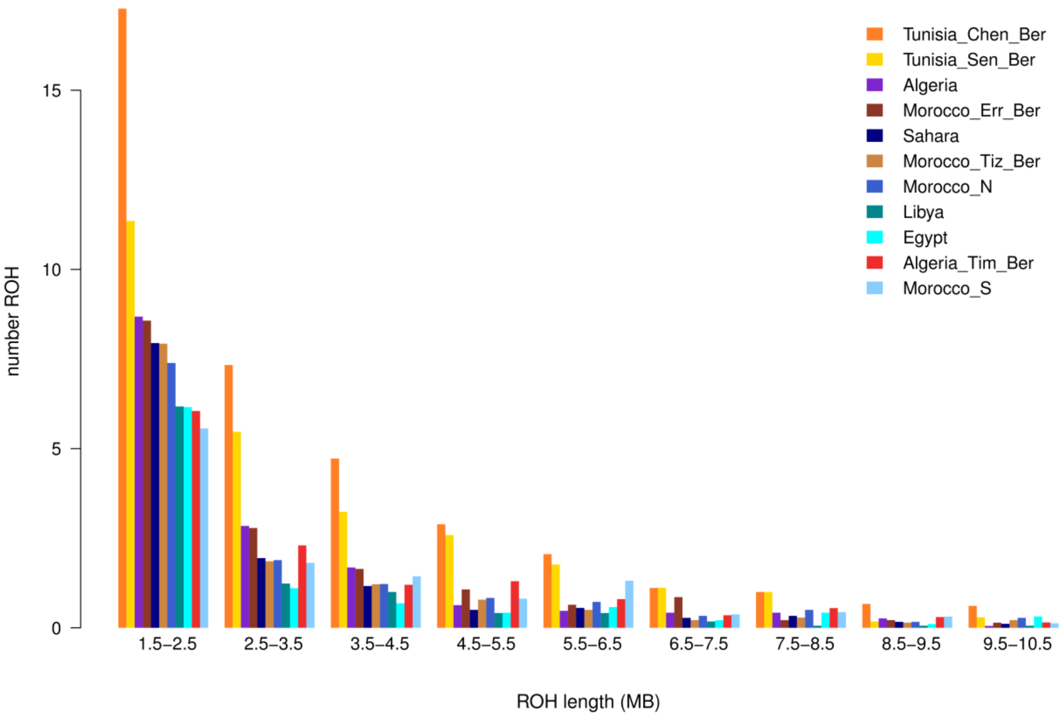

S10 Fig. Runs of homozygosity (ROH) analysis of the samples clustered by geographical origin.

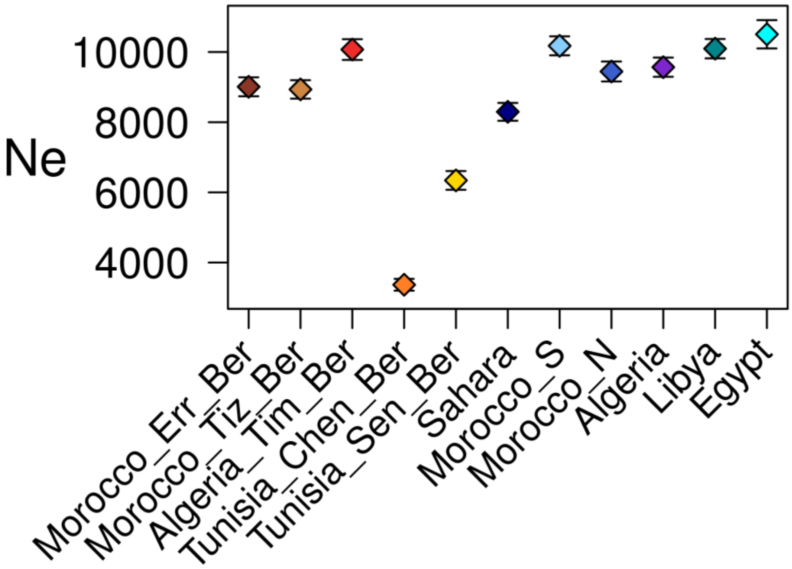

S11 Fig. Effective population size (Ne) estimated for each population.

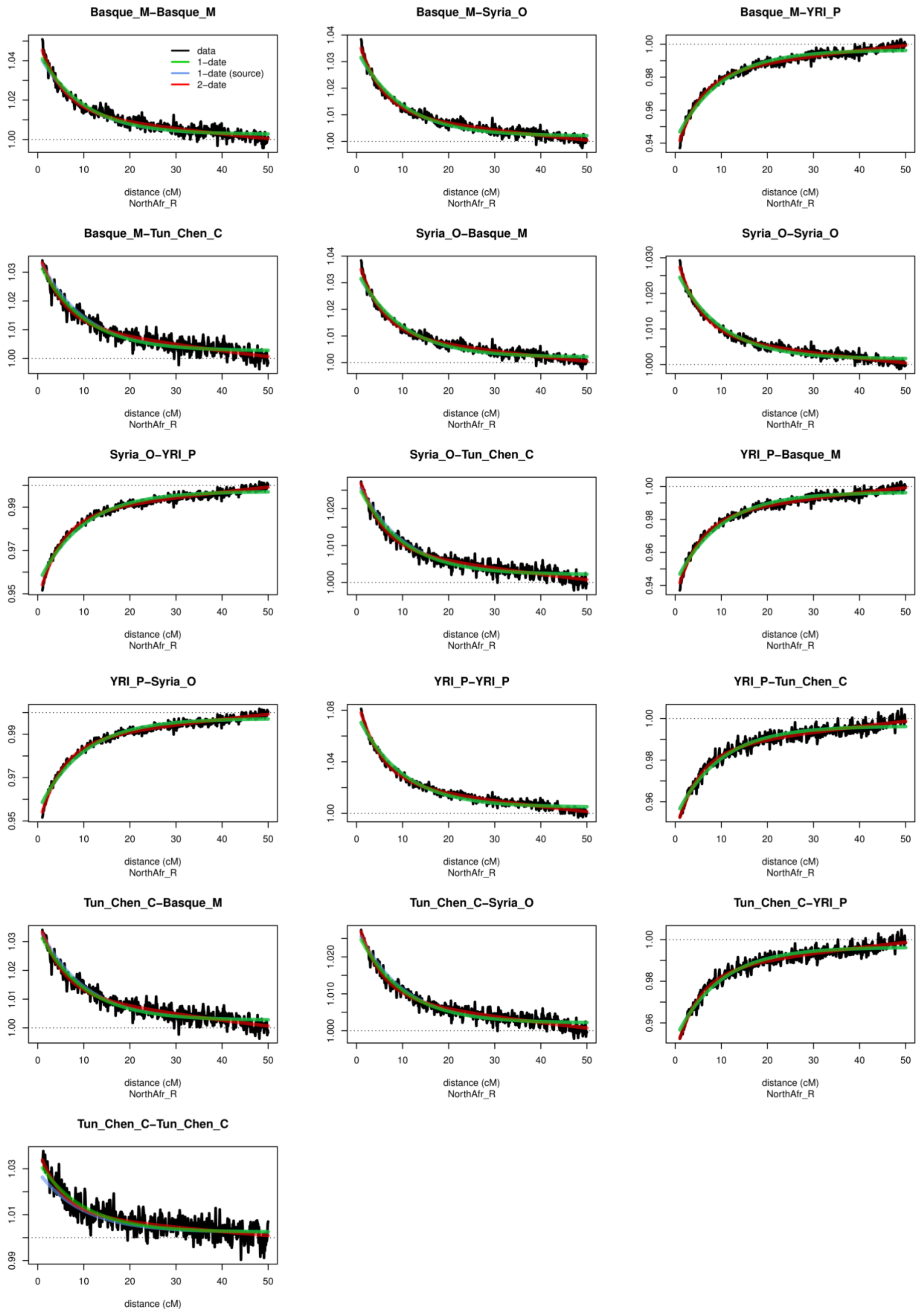

S12 Fig. Globetrotter. Globetrotter model curves estimated for cluster of individuals R.

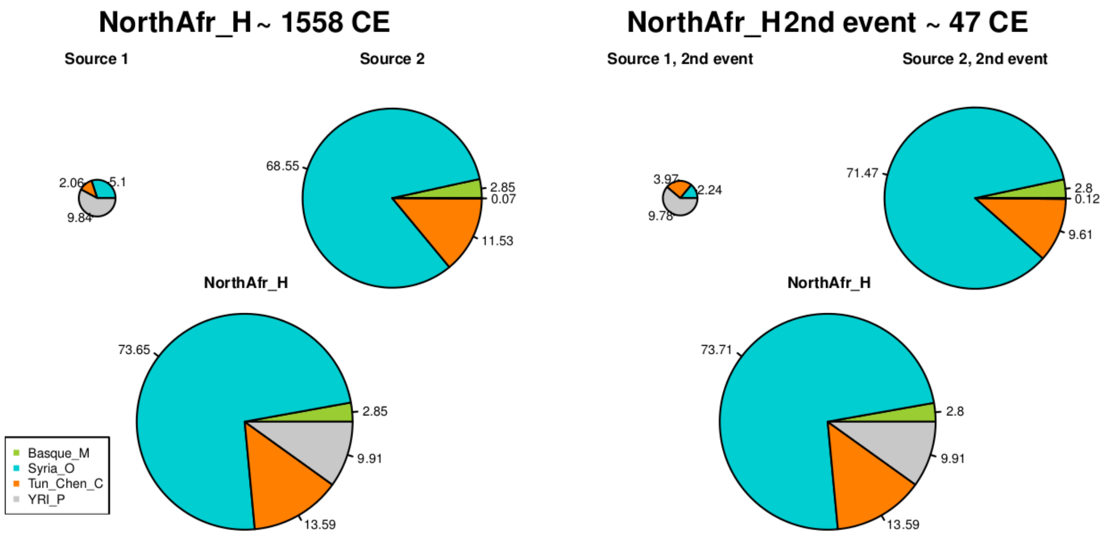

S13 Fig. Globetrotter. Example of pieplots showing the proportions of the source populations and the admixed resultant population and dates estimated with Globetrotter for cluster H.

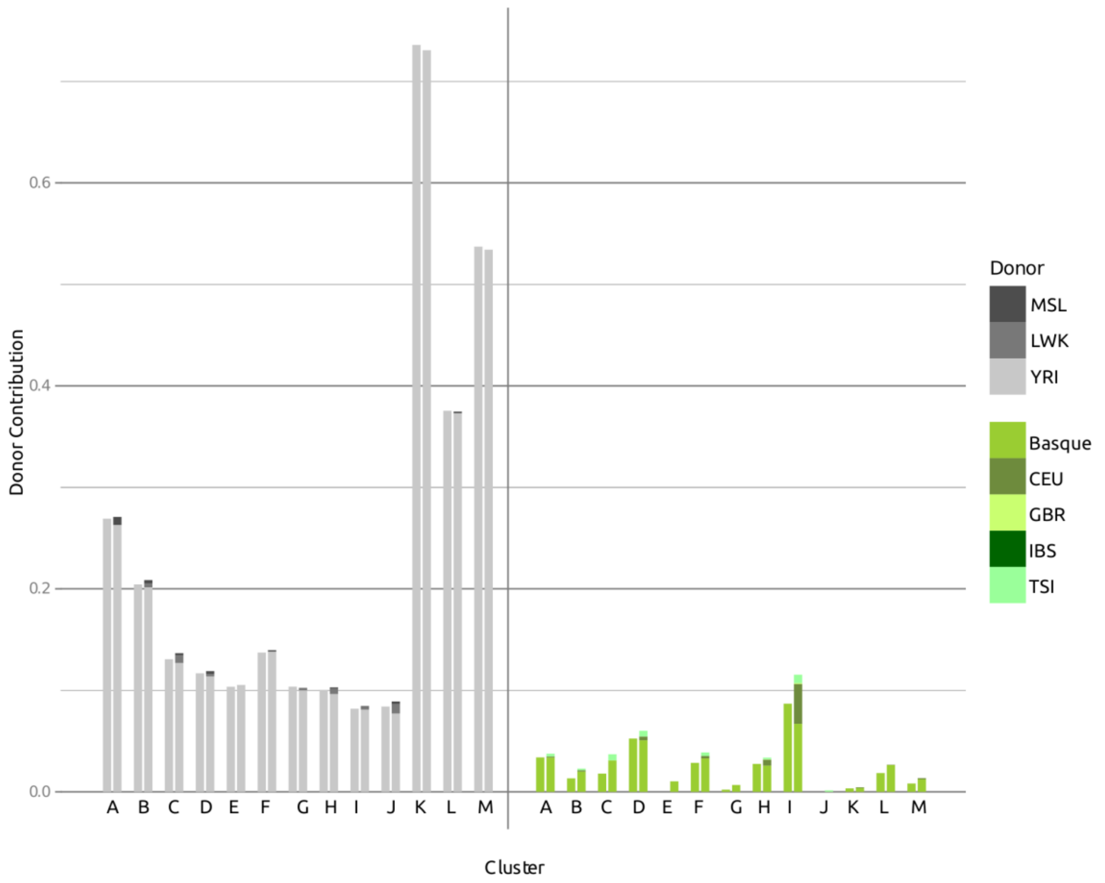

S14 Fig. Haplotype sharing proportions by cluster using different source populations. In most of the analyses, four ancestral populations were chosen (Yoruba, Basques, Syrians, and Tunisians from Chenini). In order to evaluate if the use of other ancestral proxies might affect our results, data from 1000 Genomes Phase 3 were considered. For each cluster in the x axis, the comparison between two ChromoPainter analyses is shown: the contribution of a single donor population per source region (left bar) and the contribution of multiple donor populations per source region (right bar) for sub-Saharan Africa (grayish colors) and Europe (greenish colors). No differences are found when considering extra ancestral proxies to the admixture estimations.

			I	E	G	J	C	F	H	M	K	B	D	A	L
			one-date-multiway	one-date	one-date	one-date	one-date	one-date	multiple-dates	one-date	one-date	multiple-dates	multiple-dates	multiple-dates	multiple-dates
		Mean date	1144 CE	1256 CE	1296 CE	1311 CE	1384 CE	1472 CE	1568CE	1646 CE	1670 CE	1727 CE	1745 CE	1846 CE	1936 CE
		SD (+/-)	58 years	68 years	82 years	45 years	72 years	58 years	85 years	21 years	74 years	80 years	146 years	38 years	60 years
		Median date	1148 CE	1248 CE	1292 CE	1312 CE	1373 CE	1473 CE	1568 CE	1646 CE	1668 CE	1721 CE	1766 CE	1844 CE	1972 CE
		CI 95%	1029 CE - 1255 CE	1147 CE - 1397 CE	1136 CE - 1437 CE	1213 CE - 1393 CE	1258 CE - 1520 CE	1365 CE - 1573 CE	1431 CE - 1715 CE	1604 CE - 1683 CE	1517 CE - 1807 CE	1597 CE - 1941 CE	1474 CE - 1972 CE	1780 CE - 1924 CE	1782 CE - 1972 CE
Most recent admixture events	Minor source	YRI	0.083	0.108	0.098	0.098	0.128	0.135	0.098	0.031	0.07	0.2	0,000	0.267	0.372
		Basque	0.000	0,000	0.000	0.000	0.000	0.000	0.000	0.000	0.000	0.000	0.056	0.000	0.000
		Tun.Chen.Ber.	0.036	0.012	0.006	0.000	0.038	0.022	0.021	0.076	0.044	0.025	0.126	0.041	0.026
		Syria	0.001	0.000	0.006	0.002	0.054	0.004	0.051	0.353	0.196	0.115	0.308	0.142	0.092
		Total	0.12	0.12	0.11	0.1	0.22	0.161	0.17	0.46	0.31	0.34	0.49	0.45	0.49
	Major source	YRI	0,000	0.000	0.004	0.000	0.001	0.001	0.001	0.505	0.665	0.002	0.116	0.002	0.003
		Basque	0.101	0.000	0.000	0.000	0.018	0.026	0.028	0.006	0.000	0.005	0.000	0.037	0.019
		Tun.Chen.Ber.	0.13	0.106	0.116	0.036	0.151	0.125	0.115	0.027	0.02	0.088	0.098	0.131	0.098
		Syria	0.649	0.774	0.77	0.864	0.609	0.688	0.685	0.002	0.004	0.565	0.296	0.381	0.389
		Total	0.88	0.88	0.89	0.9	0.78	0.84	0.83	0.54	0.69	0.66	0.51	0.55	0.51

									H			B	D	A	L
		Mean date							35 CE			514 CE	155 BC	643 CE	1286 CE
		SD (+/-)							501 years			441 years	723 years	273 years	304 years
		Median date							114 CE			548 CE	26 BC	635 CE	1381 CE
		CI 95%							991 BC - 774 CE			433 BC - 1330 CE	1729 BC - 797 CE	86 CE - 1101 CE	463 CE - 1531 CE
Oldest admixture events	Minor source	YRI							0.098			0.201	0.118	0.266	0.372
		Basque							0.000			0.000	0.000	0.000	0.000
		Tun.Chen.Ber.							0.04			0.043	0.086	0.091	0.036
		Syria							0.022			0.096	0.027	0.113	0.072
		Total							0.16			0.34	0.23	0.47	0.48
	Major source	YRI							0.001			0.002	0.000	0.002	0.003
		Basque							0.028			0.005	0.064	0.036	0.019
		Tun.Chen.Ber.							0.096			0.071	0.139	0.081	0.089
		Syria							0.715			0.583	0.567	0.411	0.41
		Total							0.84			0.66	0.77	0.53	0.52

S1 Table. Globetrotter results summary. Mean and standard deviations (SD in years) estimated dates, and median and CI of 95% for the different clusters are shown. Clusters with only one date event are shown in the top table, whereas multiple events are shown in both tables (top: recent events; bottom: old events).

Population	Number of individuals	Dataset	Microarray
**Luhya, Webuye, Kenya**	25	HapMap	Illumina Human1M<br>Affymetrix SNP 6.0
**Yoruba, Ibadan, Nigeria**	25	HapMap	Illumina Human1M<br>Affymetrix SNP 6.0
**Morocco North**	18	Henn et al. 2012	Affymetrix SNP 6.0
**Morocco South**	16	Henn et al. 2012	Affymetrix SNP 6.0
**Moroccan Berbers, Errachidia**	14	Current study	Affymetrix SNP 6.0
**Moroccan Berbers, Tiznit**	14	Current study	Affymetrix SNP 6.0
**Occidental Sahara**	18	Henn et al. 2012	Affymetrix SNP 6.0
**Algerian, Alger**	19	Henn et al. 2012	Affymetrix SNP 6.0
**Algerian Berbers, Timimoun**	20	Current study	Affymetrix SNP 6.0
**Algerian Mozabites Berbers**	29	HGDP	Illumina 650Y
**Tunisian Berbers,Chenini**	18	Henn et al. 2012	Affymetrix SNP 6.0
**Tunisian Berbers, Sened**	17	Current study	Affymetrix SNP 6.0
**Libya**	17	Henn et al. 2012	Affymetrix SNP 6.0
**Egypt**	19	Henn et al. 2012	Affymetrix SNP 6.0
**Bedouin, Jordan**	25	HGDP	Illumina 650Y
**Druze, Israel**	25	HGDP	Illumina 650Y
**Palestinian**	25	HGDP	Illumina 650Y
**Qatar**	25	Hunter-Zinck et al. 2010	Affymetrix 500k
**Lebanon**	24	Haber et al. 2012	Illumina 610K/ 660K
**Syria**	19	Current study	Affymetrix SNP 6.0
**Canary Islands**	17	Botigué et al. 2013	Affymetrix SNP 6.0
**Andalusian, Spain**	17	Botigué et al. 2013	Affymetrix SNP 6.0
**Galician, Spain**	17	Botigué et al. 2013	Affymetrix SNP 6.0
**Basque, Spain**	20	Henn et al. 2012	Affymetrix SNP 6.0
**Tuscan, Italy**	25	HapMap	Illumina Human1M<br>Affymetrix SNP 6.0
**Northern and Western European in Utah**	25	HapMap	Illumina Human1M<br>Affymetrix SNP 6.0
**Total**	533		

S2 Table. Dataset. Complete dataset of populations included in the analysis. For some analyses, only a subset of these populations was used in order to maximize the number of SNPs within the analysis. Berbers are highlighted in orange.

Large dataset (with HGDP samples from Middle East)	
**Step**	**Number of SNPs**
Raw data (for current study samples), all markers genotyped	909,622
Raw data (for current study samples), autosomal markers	868,366
Merged dataset filtered (missing SNPs and HWE)	17,620
Merged dataset with minor allele frequency filter	16,980
Merged dataset with LD pruning	16,185

Reduced dataset (only affymetrix 6.0 samples)	
**Step**	**Number of SNPs**
Raw data (for current study samples), all markers genotyped	909,622
Raw data (for current study samples), autosomal markers	868,366
Merged dataset filtered (missing SNPs and HWE)	165,340
Merged dataset with minor allele frequency filter	153,909
Merged dataset with LD pruning	94,905

North Africa samples with Mozabites	
**Step**	**Number of SNPs**
Raw data (for current study samples), all markers genotyped	909,622
Raw data (for current study samples), autosomal markers	868,366
Merged dataset filtered (missing SNPs and HWE)	84,450
Merged dataset with minor allele frequency filter	71,972
Merged dataset with LD pruning	67,786

North Africa samples (without Mozabites)	
**Step**	**Number of SNPs**
Raw data (for current study samples), all markers genotyped	909,622
Raw data (for current study samples), autosomal markers	868,366
Merged dataset filtered (missing SNPs and HWE)	500,237
Merged dataset with minor allele frequency filter	386,463
Merged dataset with LD pruning	200,538

Haplotype-based methods dataset	
**Step**	**Number of SNPs**
Raw data (for current study samples), all markers genotyped	909,622
Raw data (for current study samples), autosomal markers	868,366
Merged dataset filtered (missing SNPs and HWE)	327,356
Merged dataset with minor allele frequency filter	293,908

S3 Table. SNP density. SNP density for all datasets used considering the different filters applied.

	DIFFERENCES					P-VALUES				
	Cluster	YRI	Tun_Chen	Syria	Basque	Cluster	YRI	Tun_Chen	Syria	Basque
**Chr 1 vs rest of autosomes**	A	0.047	-0.014	-0.009	-0.024	A	0.011	0.235	0.896	0.098
	B	-0.013	0.008	0.008	-0.003	B	0.262	0.389	0.410	0.489
	C	-0.003	-0.010	0.028	-0.016	C	0.578	0.312	0.110	0.065
	D	-0.006	0.007	0.008	-0.009	D	0.275	0.742	0.304	0.313
	E	0.026	0.002	-0.028	0.000	E	0.052	0.908	0.089	1.000
	F	0.000	0.011	0.011	-0.022	F	0.894	0.421	0.685	0.103
	G	-0.019	0.027	-0.008	0.000	G	0.304	0.000	0.516	1.000
	H	-0.010	-0.013	0.034	-0.011	H	0.148	0.069	0.036	0.373
	I	0.001	-0.004	0.014	-0.012	I	0.868	0.286	0.202	0.306
	J	-0.003	-0.001	0.004	0.000	J	0.662	0.845	0.644	1.000
	K	-0.030	0.027	0.003	0.000	K	0.351	0.009	0.807	1.000
	L	0.024	0.011	-0.016	-0.020	L	0.415	0.436	0.607	0.119
	M	0.017	-0.002	-0.018	0.003	M	0.480	0.762	0.536	1.000
	Cluster	YRI	Tun_Chen	Syria	Basque	Cluster	YRI	Tun_Chen	Syria	Basque
**Chr 2 vs rest of autosomes**	A	-0.018	0.002	-0.033	0.050	A	0.392	0.935	0.103	0.000
	B	-0.018	0.017	-0.030	0.030	B	0.191	0.251	0.379	0.040
	C	0.023	0.004	-0.040	0.013	C	0.005	0.785	0.064	0.349
	D	0.011	0.002	-0.043	0.030	D	0.044	0.595	0.009	0.006
	E	-0.001	0.005	-0.005	0.000	E	0.555	0.453	0.715	1.000
	F	-0.014	-0.010	0.001	0.023	F	0.219	0.073	0.757	0.025
	G	-0.004	-0.041	-0.024	0.068	G	0.742	0.074	0.519	0.000
	H	0.019	-0.017	-0.020	0.017	H	0.026	0.011	0.285	0.092
	I	-0.001	-0.005	-0.027	0.034	I	0.837	0.249	0.163	0.008
	J	-0.001	-0.002	0.004	0.000	J	0.919	0.600	0.718	1.000
	K	0.003	0.009	-0.012	0.000	K	0.910	0.501	0.640	1.000
	L	0.022	-0.004	-0.049	0.030	L	0.382	0.691	0.039	0.000
	M	0.021	0.002	-0.036	0.013	M	0.312	0.815	0.081	0.354
	Cluster	YRI	Tun_Chen	Syria	Basque	Cluster	YRI	Tun_Chen	Syria	Basque
**Chr 3 vs rest of autosomes**	A	0.036	0.031	-0.109	0.042	A	0.103	0.000	0.000	0.003
	B	0.021	0.024	-0.083	0.038	B	0.346	0.038	0.000	0.003
	C	0.023	0.012	-0.070	0.035	C	0.040	0.387	0.009	0.020
	D	0.015	0.015	-0.058	0.029	D	0.000	0.129	0.003	0.034
	E	0.023	-0.003	-0.020	0.000	E	0.096	0.712	0.173	1.000
	F	0.017	0.011	-0.049	0.022	F	0.213	0.439	0.239	0.454
	G	0.035	0.008	-0.071	0.028	G	0.000	0.812	0.000	0.074
	H	0.022	0.011	-0.063	0.029	H	0.005	0.279	0.000	0.020
	I	0.023	0.011	-0.081	0.047	I	0.000	0.342	0.000	0.005
	J	-0.004	0.017	-0.013	0.000	J	0.616	0.000	0.149	1.000
	K	-0.029	0.005	0.016	0.008	K	0.072	0.031	0.289	0.761
	L	-0.025	0.023	-0.001	0.004	L	0.527	0.056	0.894	0.817
	M	0.043	0.002	-0.052	0.008	M	0.063	0.918	0.016	0.699
	Cluster	YRI	Tun_Chen	Syria	Basque	Cluster	YRI	Tun_Chen	Syria	Basque
**Chr 4 vs rest of autosomes**	A	-0.013	0.020	-0.013	0.005	A	0.479	0.021	0.738	0.912
	B	-0.026	0.008	0.021	-0.002	B	0.137	0.518	0.181	0.577
	C	-0.004	0.004	0.010	-0.009	C	0.573	0.846	0.622	0.318
	D	0.005	0.016	0.005	-0.026	D	0.228	0.159	0.415	0.008
	E	0.017	0.003	-0.019	0.000	E	0.042	0.779	0.099	1.000
	F	0.058	0.026	-0.104	0.021	F	0.002	0.053	0.000	0.486
	G	-0.006	0.032	-0.025	0.000	G	0.525	0.000	0.000	1.000
	H	-0.007	-0.006	0.037	-0.025	H	0.365	0.479	0.065	0.021
	I	0.001	-0.001	0.002	-0.003	I	0.823	0.711	0.643	0.579
	J	0.001	0.000	0.000	0.000	J	0.858	0.901	0.943	1.000
	K	0.037	-0.004	-0.038	0.004	K	0.040	0.542	0.238	0.649
	L	0.002	0.000	0.020	-0.021	L	0.917	0.781	0.331	0.000
	M	-0.015	0.005	-0.007	0.017	M	0.525	0.750	0.935	0.195

	DIFFERENCES					P-VALUES				
	Cluster	YRI	Tun_Chen	Syria	Basque	Cluster	YRI	Tun_Chen	Syria	Basque
Chr 5 vs rest of autosomes	A	-0.068	0.007	0.079	-0.018	A	0.004	0.776	0.049	0.388
	B	-0.020	-0.015	0.029	0.006	B	0.266	0.106	0.383	0.919
	C	0.015	0.002	-0.025	0.009	C	0.111	0.949	0.222	0.790
	D	0.002	-0.020	0.021	-0.003	D	0.785	0.002	0.135	0.609
	E	0.001	-0.018	0.017	0.000	E	0.911	0.031	0.214	1.000
	F	-0.028	-0.006	0.044	-0.010	F	0.022	0.506	0.003	0.315
	G	-0.025	0.000	0.024	0.000	G	0.000	0.815	0.300	1.000
	H	-0.030	0.000	0.017	0.013	H	0.000	0.899	0.208	0.392
	I	-0.019	-0.015	0.009	0.025	I	0.001	0.019	0.448	0.109
	J	-0.027	0.000	0.027	0.000	J	0.000	0.978	0.002	1.000
	K	0.021	-0.006	-0.015	0.000	K	0.641	0.637	0.637	1.000
	L	0.008	-0.022	0.011	0.003	L	0.873	0.171	0.649	0.998
	M	0.017	-0.012	-0.009	0.004	M	0.622	0.203	0.856	0.963
	Cluster	YRI	Tun_Chen	Syria	Basque	Cluster	YRI	Tun_Chen	Syria	Basque
Chr 6 vs rest of autosomes	A	-0.053	0.029	0.040	-0.015	A	0.037	0.040	0.092	0.179
	B	0.023	0.010	-0.045	0.011	B	0.206	0.452	0.161	0.556
	C	-0.009	0.025	-0.029	0.013	C	0.080	0.027	0.332	0.525
	D	0.002	0.038	-0.060	0.020	D	0.650	0.001	0.000	0.165
	E	0.008	-0.011	0.004	0.000	E	0.735	0.322	0.773	1.000
	F	-0.004	0.030	-0.053	0.027	F	0.783	0.031	0.000	0.183
	G	-0.017	0.001	0.015	0.000	G	0.592	0.742	0.592	1.000
	H	-0.002	0.012	-0.040	0.030	H	0.735	0.298	0.072	0.022
	I	-0.006	0.030	-0.018	-0.006	I	0.317	0.004	0.482	0.502
	J	0.011	0.005	-0.017	0.000	J	0.098	0.171	0.024	1.000
	K	0.028	0.001	-0.041	0.012	K	0.331	0.932	0.108	0.084
	L	0.019	0.015	-0.017	-0.018	L	0.493	0.296	0.447	0.316
	M	-0.005	0.015	-0.020	0.010	M	0.712	0.246	0.473	0.670

S4 Table. Comparison in the ancestry estimation between different autosomes. In the left side the values for the comparisons between each chromosome from 1 to 6 vs the rest of autosomes are shown. The p-values for the corresponding comparisons are shown in the right side. Significant p-values are highlighted in orange.
